# Early ART reduces viral seeding and innate immunity in liver and lungs of SIV-infected macaques

**DOI:** 10.1172/jci.insight.167856

**Published:** 2023-07-24

**Authors:** Julien A. Clain, Henintsoa Rabezanahary, Gina Racine, Steven Boutrais, Calaiselvy Soundaramourty, Charles Joly Beauparlant, Mohammad-Ali Jenabian, Arnaud Droit, Petronela Ancuta, Ouafa Zghidi-Abouzid, Jérôme Estaquier

**Affiliations:** 1 CHU de Québec Research Center, Laval University, Quebec City, Quebec, Canada.; 2 INSERM U1124, University of Paris, Paris, France.; 3 Department of Biological Sciences and CERMO-FC Research Centre, University of Quebec in Montreal, Montreal, Quebec, Canada.; 4 Research Center of the University of Montreal Hospital Center, Montreal, Quebec, Canada.; 5 Department of Microbiology, Infectiology, and Immunology, Faculty of Medicine, University of Montreal, Montreal, Quebec, Canada.

**Keywords:** AIDS/HIV, Innate immunity, Macrophages

## Abstract

Identifying immune cells and anatomical tissues that contribute to the establishment of viral reservoirs is of central importance in HIV-1 cure research. Herein, we used rhesus macaques (RMs) infected with SIVmac251 to analyze viral seeding in the liver and lungs of either untreated or early antiretroviral therapy–treated (ART-treated) RMs. Consistent with viral replication and sensing, transcriptomic analyses showed higher levels of inflammation, pyroptosis, and chemokine genes as well as of interferon-stimulating gene (ISG) transcripts, in the absence of ART. Our results highlighted the infiltration of monocyte-derived macrophages (HLA-DR^+^CD11b^+^CD14^+^CD16^+^) in inflamed liver and lung tissues associated with the expression of CD183 and CX3CR1 but also with markers of tissue-resident macrophages (CD206^+^ and LYVE^+^). Sorting of myeloid cell subsets demonstrated that CD14^+^CD206^–^, CD14^+^CD206^+^, and CD14^–^CD206^+^ cell populations were infected, in the liver and lungs, in SIVmac251-infected RMs. Of importance, early ART drastically reduced viral seeding consistent with the absence of ISG detection but also of genes related to inflammation and tissue damage. Viral DNA was only detected in CD206^+^HLA-DR^+^CD11b^+^ cells in ART-treated RMs. The observation of pulmonary and hepatic viral rebound after ART interruption reinforces the importance of early ART implementation to limit viral seeding and inflammatory reactions.

## Introduction

The observation that viral rebound occurs after antiretroviral therapy (ART) interruption (ATI) despite early treatment indicated early viral seeding and the absence of the full eradication of HIV and SIV (1–4). Persistence of proviral DNA in HIV/SIV-infected cells is considered one of the main impediments to eradicate the virus. Therefore, the identification of cellular and anatomic viral reservoirs is a major challenge in finding a cure for HIV (5).

Monocytes play a critical role in tissue homeostasis and host defense (6). Blood monocytes that develop in the bone marrow are released in the circulation as nondividing cells. Monocyte subsets are defined as classical, intermediate, and nonclassical cells, based on the expression of CD14 and CD16 molecules (7). Monocytes continuously replenish the intestine and the spleen and are mobilized into inflammatory sites, in response to tissue injuries (8, 9). Once the monocytes home to inflamed tissues, they can differentiate into inflammatory macrophages (M1, classical) and exert antimicrobial activity (10). Thus, monocytes have been demonstrated to develop a strong plasticity, involved not only in inflammation but also in tissue repair (11–13). We, and others, have also shown that monocytes are infected (14–16) and that ART prevents the infection of monocytes (3).

In addition to monocyte-derived macrophages (10), tissue-resident macrophages (TRMs), which are derived from yolk sac, are long-lived, self-renewing TRMs colonizing the brain (microglia), liver (Kupffer cells), and lung (alveolar macrophages) (17, 18). However, other groups have challenged the notion of long-lived TRM cells (19, 20). Whereas several markers have been previously proposed to characterize TRMs, mostly in the mouse model conducted in healthy animals during homeostasis, little is known so far about their expressions in SIV-infected rhesus macaques (RMs). These markers included the hyaluronan receptors (CD44 and LYVE-1, lymphatic vessel endothelial receptor 1) (21–23), the phosphatidylserine receptor TIM-4 (T cell immunoglobulin and mucin domain containing 4) (24, 25), CD117 (c-KIT, a type III receptor tyrosine kinase) (26), CD206 (a mannose receptor C type 1) (27) described in the hearts of RMs (28), CD200R (an inhibitory receptor) (29), and CD64 (an Fc-γ receptor 1, FcγRI) (26, 30). However, the definition of macrophage populations in the context of damaged tissues or inflammation is more complex. Thus, lung fibrosis is associated with the differentiation of monocytes into alveolar macrophages persisting over the life span (31), whereas monocyte-derived macrophages infiltrating inflamed tissue can also acquire TRM markers (TRM-like cells) (32–34). Furthermore, the colony stimulating factor (CSF1) plays a role in the recruitment and differentiation of inflamed macrophages into resident macrophages after injury (19, 20), adding another level of heterogeneity encompassing distinct macrophage populations.

We demonstrated that in inflamed lung and liver tissues of SIV-infected RMs, CD206^+^ and LYVE^+^ macrophages also express CD183 (CXCL10 or IP-10 receptor), suggesting recruitment consistent with higher levels of chemokine and innate immune transcripts. In particular, genes related to inflammasome/pyroptosis and tissue damage are increased (35). By sorting CD11b^+^HLA-DR^+^ cell subsets, excluding CD3^+^CD20^+^cells, we demonstrated that CD14^+^CD206^–^, CD14^+^CD206^+^, and CD14^–^CD206^+^ populations contained viral DNA, as well, in CD4^+^ T cells. Administration of early ART drastically reduced viral seeding, inflammation, and innate immune response, both in hepatic and in pulmonary tissues. However, despite early ART, altered expression of metabolic gene signature persisted in the lungs that can be related to the presence of viral DNA in CD11b^+^HLA-DR^+^CD206^+^ cells.

## Results

### Viral DNA and RNA in the liver and lungs of SIV-infected RMs.

**Figure 1:**
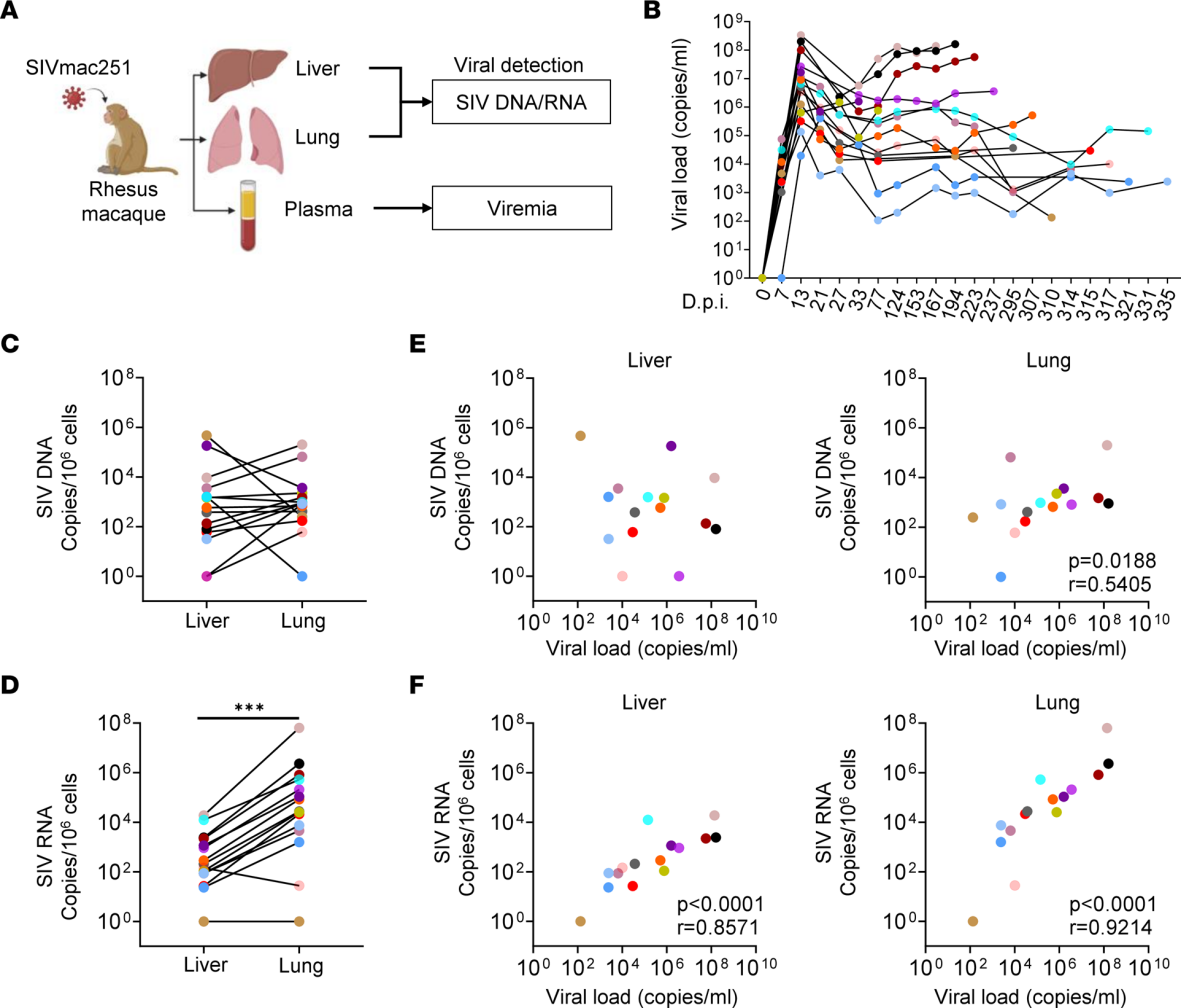
Detection of cell-associated SIV DNA and RNA in the liver and lungs of SIV-infected RMs. (**A**) Schematic of viral quantifications in the liver, lungs, and plasma of SIV-infected RMs, created with BioRender.com. (**B**) Longitudinal plasma viral loads in untreated SIV-infected RMs. (**C**) SIV DNA and (**D**) SIV RNA from the liver and lungs of SIV-infected RMs were quantified by qRT-PCR. Viral load of SIV-infected RMs was correlated against frequencies of (**E**) SIV DNA and (**F**) SIV RNA. Spearman’s analysis was used for correlations. The *r* and *P* values are indicated in the figures. Each colored symbol represents 1 individual (*n* = 15). Cell-associated SIV DNA and RNA are expressed as copies per 10^6^ cells. A 1-tailed Wilcoxon’s test was performed; *** indicates *P* < 0.001.

RMs of Chinese origin were infected with SIVmac251 and sacrificed at different time points, postinfection (Figure 1A and Supplemental Table 1; supplemental material available online with this article; https://doi.org/10.1172/jci.insight.167856DS1). During infection, plasma viral loads reached 10^2^ to 10^8^ copies/mL (Figure 1B). We then assessed viral seeding in the liver and lungs of SIV-infected RMs. The amount of viral DNA quantified by quantitative real-time PCR (qRT-PCR) in the liver of untreated SIV-infected RMs ranged from 6 × 10^1^ to 4.7 × 10^5^ copies/10^6^ cells, whereas viral DNA in the lungs ranged from 1.7 × 10^2^ to 2 × 10^5^ copies/10^6^ cells (Figure 1C). Thus, no significant differences were observed between the amounts of viral DNA in the 2 organs. In contrast to viral DNA, we found that the levels of viral RNA (ranging from 2.3 × 10^1^ to 1.9 × 10^4^ copies/10^6^ cells) were lower in the liver, compared with the levels of viral RNA in the lungs (ranging from 2.8 × 10^1^ to 6.3 × 10^7^ copies/10^6^ cells, *P* = 0.0004) (Figure 1D). We then assessed the correlation between plasma viral loads (VLs) and the levels of viral DNA and RNA. Whereas no correlation was observed between VL and the amount of viral DNA (Figure 1E), a strong positive correlation was observed between VL and viral RNA, both in the liver (Spearman’s *P* < 0.0001, *r* = 0.8571) and the lungs (Spearman’s *P* < 0.0001, *r* = 0.9214) (Figure 1F). We then assessed the extent of viral replication in both tissues after ART administrated at day 4 postinfection (Supplemental Figure 1A). Associated with an extremely low (Supplemental Figure 1B) or undetectable plasma viremia at the date of sacrifice (Supplemental Figure 1C), viral DNA and RNA were absent both in the liver and lungs (Supplemental Figure 1, D and E).

Thus, our results are consistent with previous reports, showing viral DNA and RNA in the lungs (36–39) and liver (40–43) of SIV-infected RMs in which early ART drastically reduced viral dissemination.

### Transcriptomic analyses differentiate viremic and ART-treated RMs.

Next, we performed transcriptomic analyses from the liver and lung cells of untreated SIVmac251-infected RMs including highly viremic RMs (PB023, PB028, PB044, and PB013) and low viremic RMs (12-2070R, 13-1054R, 12-1688R, and 13-1596R) (Supplemental Figure 2A). Transcriptomic analyses were also performed using healthy uninfected RMs (PB057, PB061, and PB067) and ART-treated RMs (R110562, R110360, and 12-1836R).

**Figure 2:**
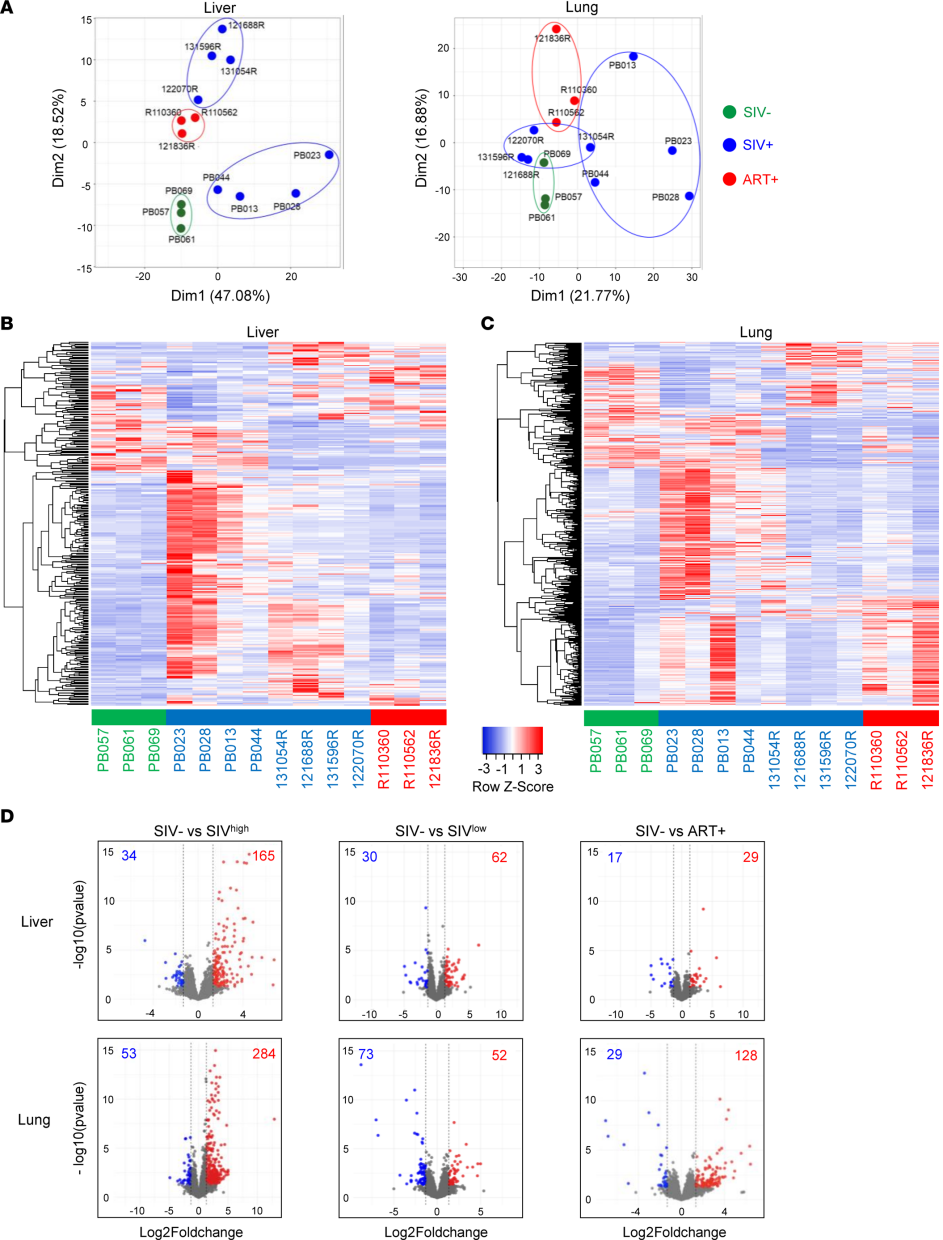
Transcriptomic signature of liver and lung tissues of naive, SIV-infected, and ART-treated RMs. (**A**) Principal component analysis of liver and lung gene expression profiles of uninfected (green symbols, *n* = 3), SIV-infected (blue symbols, *n* = 8), and ART-treated (red symbols, *n* = 3) individuals. Each data point corresponds to the set of gene expression RNA-Seq read counts. The names of each individual are indicated next to each point. The heatmaps show the differential expression of (**B**) liver and (**C**) lung genes between naive RMs, SIV-infected RMs, and ART-treated RMs. Row *z* score shows the differential expression of a single gene across the samples. Red bars indicate an increased abundance of the corresponding genes, and blue bars indicate a decreased abundance. (**D**) Volcano plots show the distribution of differentially expressed genes between naive RMs, SIV-infected RMs, and ART-treated RMs. Red plots represent upregulated genes and blue plots represent downregulated genes. *P* value ≤ 0.05 was used as the threshold for the significance of the difference in gene expression. SIV^hi^, highly viremic SIV; SIV^lo^, low viremic SIV.

The profiles of transcripts in both tissues were compared using principal component analysis (PCA) (Figure 2A). PCA, along the dimension 1 and 2 axes, discriminated healthy uninfected RMs (green) and ART-treated RMs (red) from SIV-infected RMs (blue). In the liver, we observed a clustering in 2 groups of SIV-infected RMs, reflecting the extent of viremia (highly and low viremic RMs, Supplemental Figure 2A). Of interest, the low viremic group was distinct from naive and closer to ART-treated RMs. In the lungs, a similar cluster was observed, except for 13-1054R, which was closer to highly viremic RMs (Figure 2A). The heatmap shows the rows *z* score scaled (Figure 2, B and C), indicating genes that were modulated, providing strong differential hepatic and pulmonary signatures of SIV-infected RMs compared with healthy uninfected RMs. The highest viremic RMs displayed distinct hierarchical clustering of differentially expressed genes compared with the lowest viremic animals, distinct from naive and ART-treated RMs (Figure 2, B and C).

Given the difference in highly viremic (SIV^hi^) and low viremic (SIV^lo^) RMs, we then performed additional analyses separating these 2 groups. Volcano plots showed the number of transcripts that were significantly up- and downregulated (Figure 2D, and the lists of genes are in Supplemental Table 2, A and B). Thus, compared with healthy uninfected RMs, 34 and 53 genes were downregulated in the liver and lungs, respectively, of SIV^hi^-infected RMs, whereas 30 and 73 genes were downregulated in the liver and lungs of SIV^lo^-infected RMs (Figure 2D). We also observed that 17 and 29 genes decreased in ART-treated RMs compared with healthy uninfected RMs (Figure 2D). Furthermore, our analysis revealed higher levels of genes expressed in SIV^hi^-infected RMs compared with SIV^lo^-infected RMs consistent with the heatmap. Thus, in SIV^hi^-infected RMs, 165 and 284 genes were significantly upregulated in the liver and lungs, while only 62 and 52 genes were overexpressed in the lungs and liver of SIV^lo^-infected RMs compared with healthy uninfected RMs (Figure 2D). ART administration drastically reduced the number of genes upregulated in the liver, since only 29 genes were identified compared with healthy uninfected RMs (Figure 2D). However, our results highlighted that despite early ART, the number of genes upregulated in the lungs, 128, compared with healthy uninfected RMs remained elevated (Figure 2D).

Thus, transcriptomic analyses differentiated SIV^hi^- and SIV^lo^-infected animals, demonstrating that, despite early ART, an altered pulmonary profile persists in SIV-infected RMs.

### Innate immune defense and tissue injury profiles characterize viremic SIV-infected RMs.

Having observed differentially expressed genes in both tissues from SIV^hi^- and SIV^lo^-infected RMs, we then analyzed the nature of the expressed genes. Functional enrichment analyses demonstrated that upregulated genes in SIV^hi^ RMs were associated with immune defense response pathway (Gene Ontology [GO]: 0006952: liver, *P* = 7.686 × 10^–69^; lungs, *P* = 1.854 × 10^–85^) and cell activation pathway (GO: 0001775, liver, *P* = 3.916 × 10^–12^; lungs, *P* = 5.390 × 10^–5^) (Supplemental Figure 2, B and C). In the lowest viremic RMs (SIV^lo^), upregulated genes were associated with immune defense response pathway in the liver (GO: 0006952, *P* = 6.904 × 10^–12^) and mostly associated with chemical homeostasis pathway in the lungs (GO: 0048878, *P* = 1.349 × 10^–5^). The lists of genes are indicated in Supplemental Table 3, A and B.

**Figure 3:**
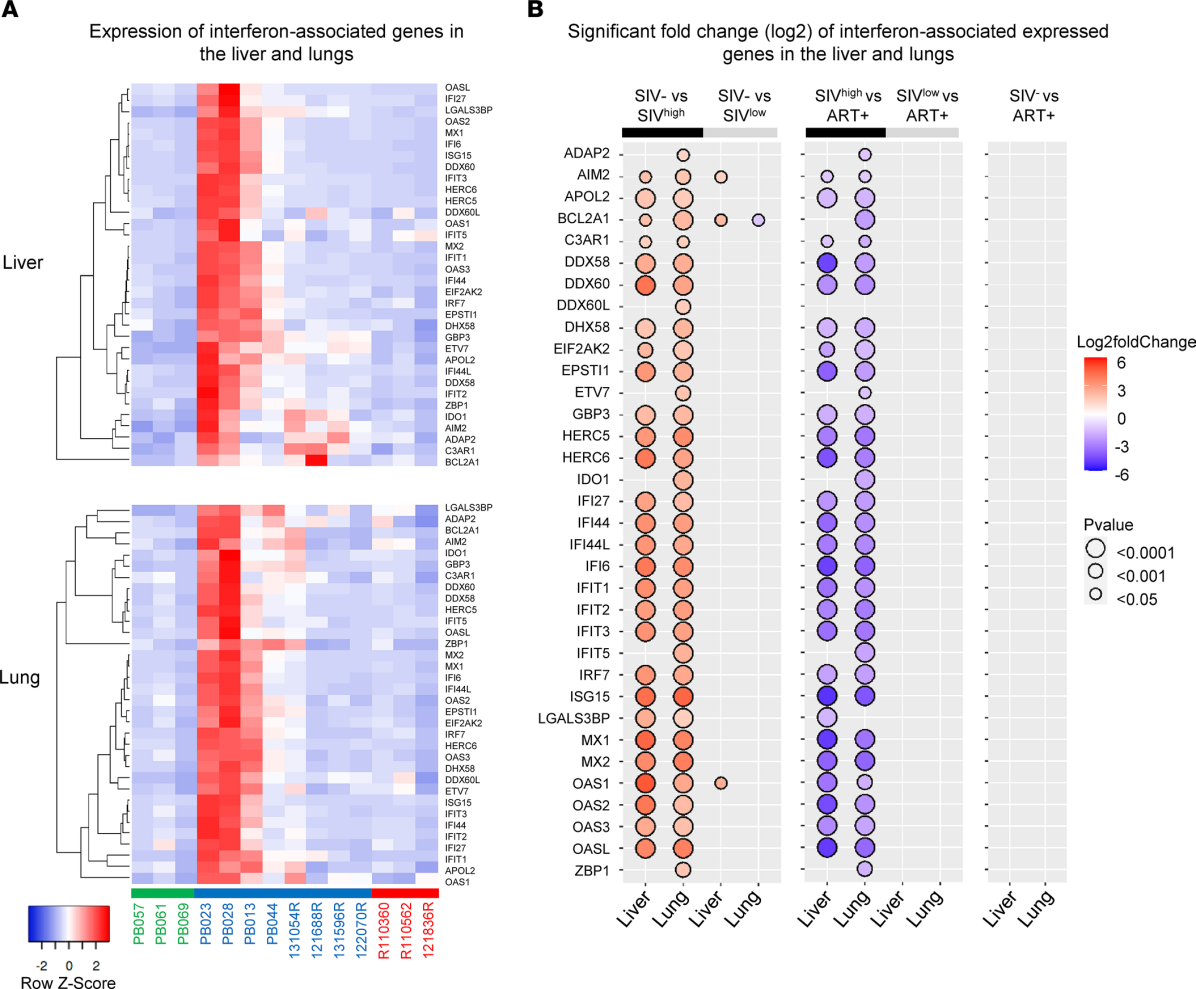
Expression of interferon-associated genes in the liver and lungs of untreated SIV-infected and ART-treated RMs. (**A**) Heatmaps show the differential expression of interferon-associated genes in the liver and lungs between naive RMs (*n* = 3), SIV-infected RMs (*n* = 8), and ART-treated RMs (*n* = 3). Row *z* score shows the differential expression of a single gene across the samples. Red bars indicate an increased abundance of the corresponding genes, and blue bars indicate a decreased abundance. (**B**) Significant log_2_ fold-change of interferon transcripts between i) naive and SIV-infected RMs (left panel), ii) SIV-infected and ART-treated RMs (middle panel), and iii) naive and ART-treated RMs (right panel). The bidirectional color-coded bubbles represent the log_2_ fold-change *z* score, whereas the size of the bubbles indicates the –log_10_ (*P* value) with a threshold of *P* < 0.0001 for highly significant values. SIV^hi^, highly viremic SIV; SIV^lo^, low viremic SIV.

Consistent with viral sensing (immune defense response), significant enrichment of interferon-stimulating gene (ISG) mRNA, including *ISG15*, *IFI6/27/44/44L* (interferon-α–inducible proteins), *IFIT1/2/3/5* (interferon-induced proteins with tetratricopeptide repeats), *IRF7* (interferon regulatory factor 7), *OAS1/2/3/L* (2′-5′-oligoadenylate synthetase), and *MX1/2* (MX dynamin like GTPase), and several ISG-related transcripts was observed in SIV^hi^-infected RMs, compared with SIV^lo^-infected RMs and healthy uninfected RMs (Figure 3A). The *P* values and log_2_ fold-changes, associated with up- and downregulated genes, are represented in Figure 3B and listed in Supplemental Table 2, A and B. Furthermore, ART administration drastically reduced their expression, leading to the absence of enrichment of these genes in ART-treated SIV-infected, compared with healthy uninfected, RMs (Figure 3B). By performing qRT-PCR of several ISGs (*ISG15*, *OAS1*, *IRF7*, and *MX1* genes), we demonstrated a positive correlation between the levels of mRNA transcripts and VL (Supplemental Figure 3), with *OAS1* being correlated only in the lungs. The absence of ISG detection was consistent with the absence of viral detection (44).

**Figure 4:**
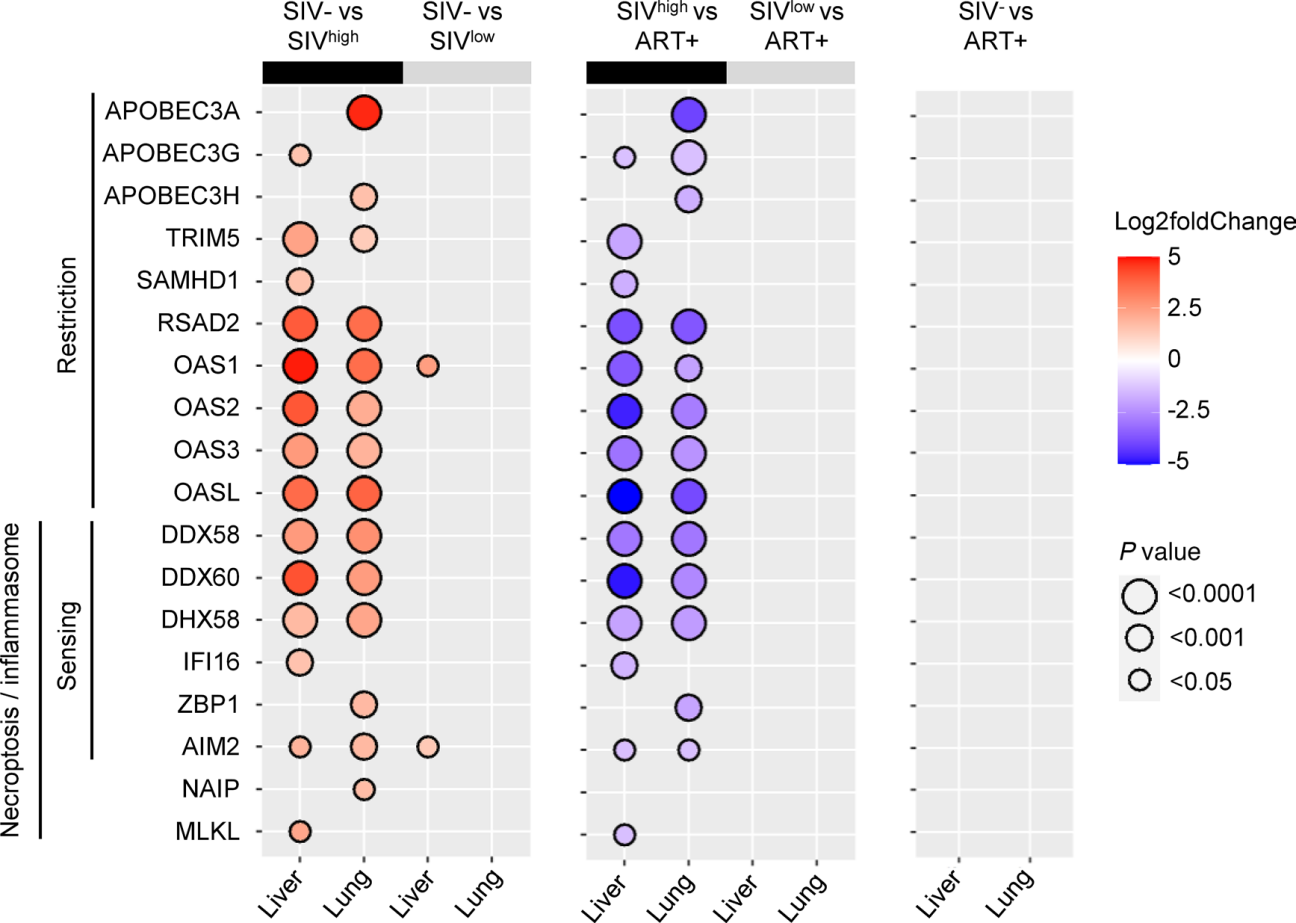
Expression of viral sensor–associated genes in the liver and lungs of untreated SIV-infected and ART-treated RMs. Significant log_2_ fold-change of transcripts related to viral restriction, viral RNA/DNA sensing, and necroptosis/inflammasome between i) naive and SIV-infected RMs (left panel), ii) SIV-infected and ART-treated RMs (middle panel), and iii) naive and ART-treated RMs (right panel). The bidirectional color-coded bubbles represent the log_2_ fold-change *z* score, whereas the size of the bubbles indicates the –log_10_ (*P* value) with a threshold of *P* < 0.0001 for highly significant values. SIV^hi^, highly viremic SIV; SIV^lo^, low viremic SIV.

Furthermore, related to the immune defense response, several HIV restriction factors, such as tripartite motif containing 5 (*TRIM5*) and *Viperin* (*RSAD2*), were also increased in both tissues of SIV^hi^-infected RMs compared with healthy uninfected RMs and SIV^lo^-infected RMs (Figure 4). Whereas *APOBEC3G* and *SAMHD1* gene transcripts were upregulated in the liver (Figure 4 and Supplemental Table 2, A and B), *APOBEC3A* and *APOBEC3H* were increased in the liver. We also observed higher levels of transcripts encoding viral RNA sensors such as *DDX58* (*RIG-1*), *DDX60*, and *DHX58* (*LGP2*) in the liver and lungs of SIV^hi^-infected RMs compared with healthy uninfected and SIV^lo^-infected RMs (Figure 4). We also found higher levels of genes related to the inflammasome and pyroptosis pathways that may contribute to tissue damage (35, 45) such as *NAIP*, *MLKL*, *IFI16*, as well as *ZBP1* (*DAI*) and *AIM2* (Figure 4). Consistent with the absence of viral detection in the tissues, all of them were downregulated after ART, and no difference was observed between ART-treated SIV-infected and healthy uninfected RMs (Figure 4).

Similar to tissue damage, we observed that transcripts related to the extracellular matrix organization (GO: 0030198: liver, *P* = 7.396 × 10^–5^; lungs, *P* = 8.459 × 10^–17^) were modulated in SIV-infected RMs (Supplemental Figure 4). Most of the altered genes related to proteolysis (GO: 0006508, *P* = 3.770 × 10^–22^) were observed in the lungs. Among them, we found that several members of the collagen family and metalloproteinases were downregulated. In contrast, several serine protease inhibitors, including members of the serpin family (*SERPINA1*, *SERPINA3*, *SERPINA5*, *SERPINA6*, *SERPINA7*, *SERPINC1*, and *SERPINF2*) and of the inter-alpha-trypsin inhibitor (*ITIH*) family (*ITIH1*, *ITIH2*, and *ITIH4*) were mostly upregulated in SIV^hi^-infected RMs (Supplemental Figure 4 and Supplemental Table 2B). Of importance, our results demonstrated the persistence of an altered pulmonary gene signature in ART-treated RMs, including members of the fibrinogen family (*FGG* and *FGL1*), *ITIH1/4*, *MMP9*, and *SERPIN*s (Supplemental Figure 4). These genes are generally observed in the context of lung tissue damage and involved in the regulation of the inflammatory response, such as in chronic obstructive pulmonary disease (COPD) and, more recently, in the context of COVID-19 (46).

Together, these results indicate that the innate immune defense response is strongly induced in the liver and lungs of the more viremic SIV-infected RMs, in which pulmonary genes associated with tissue damage persist, despite early ART.

### M1 macrophage transcripts are upregulated in the context of an altered inflammatory and metabolic environment.

**Figure 5:**
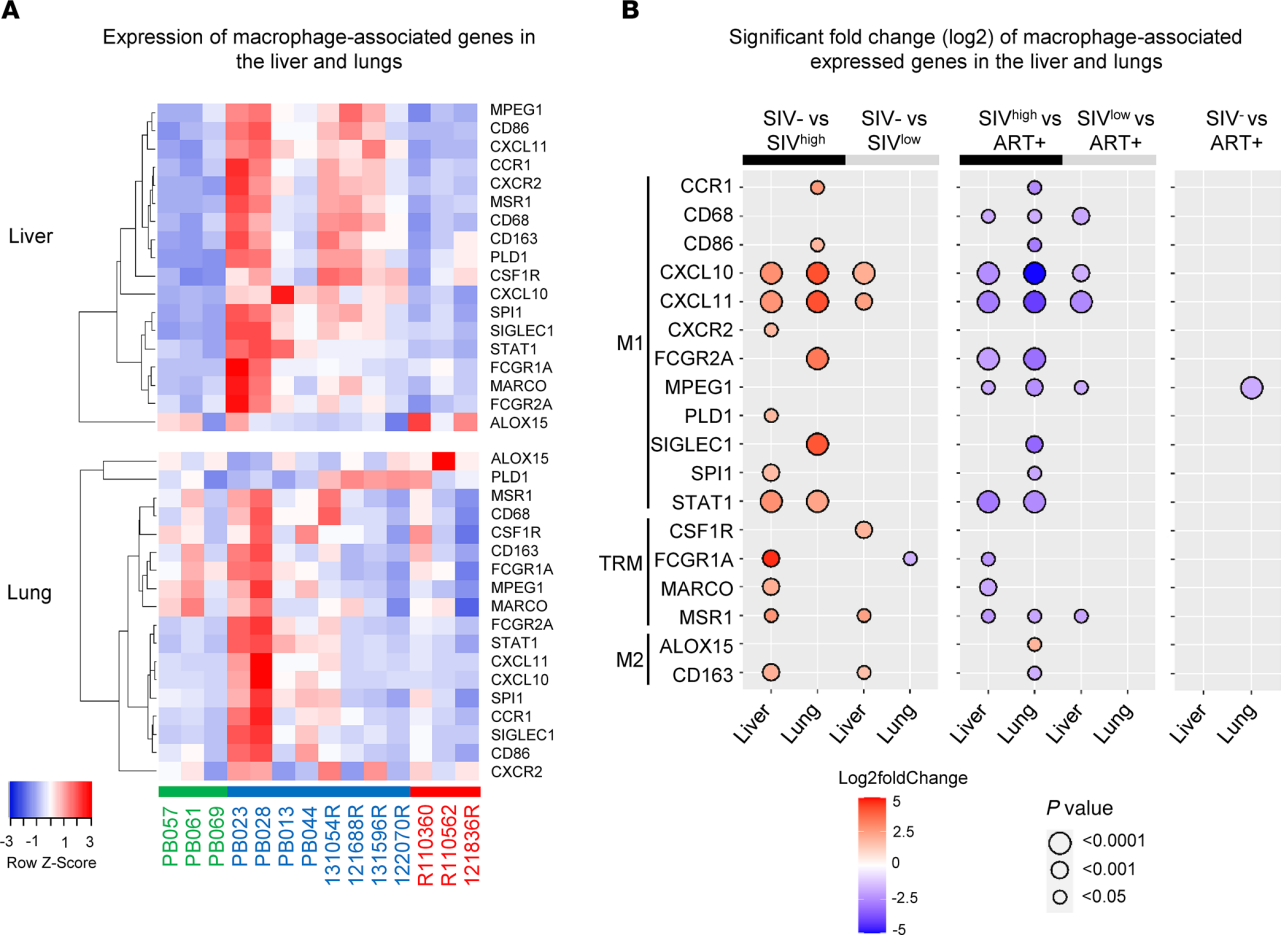
Expression of macrophage-associated genes in the liver and lungs of untreated SIV-infected and ART-treated RMs. (**A**) Heatmaps show the differential expression of macrophage-associated genes in the liver and lungs between naive RMs (*n* = 3), SIV-infected RMs (*n* = 8), and ART-treated RMs (*n* = 3). Row *z* score shows the differential expression of a single gene across the samples. Red bars indicate an increased abundance of the corresponding genes, and blue bars indicate a decreased abundance. (**B**) Significant log_2_ fold-change of macrophage (M1, TRM, M2) transcripts between i) naive and SIV-infected RMs (left panel), ii) SIV-infected and ART-treated RMs (middle panel), and iii) naive and ART-treated RMs (right panel). The bidirectional color-coded bubbles represent the log_2_ fold-change *z* score, whereas the size of the bubbles indicates the –log_10_ (*P* value) with a threshold of *P* < 0.0001 for highly significant values. SIV^hi^, highly viremic SIV; SIV^lo^, low viremic SIV.

Because tissue damage may contribute to the recruitment of inflammatory macrophages (36, 47, 48), we then analyzed transcripts related to myeloid cells. Transcriptomic analyses showed an increase of macrophage-associated genes, both in the liver and lungs (Figure 5A). We observed that most of the transcripts were associated with M1 macrophages, such as *STAT1*, *CXCL10*, and *CXCL11*, both in the liver and lungs of SIV^hi^-infected RMs (Figure 5B and Supplemental Table 2, A and B). Additional genes characterizing M1 macrophages (49), which include *CXCR2*, *PLD1*, and *SPI1*, were also upregulated in the liver, while *CCR1*, *CD86*, *FCGR2A* (CD32), and *SIGLEC1* were upregulated in pulmonary tissue (Figure 5B). Interestingly, we also observed that several transcripts reported to characterized Kupffer cells in humans were increased only in the liver, including *MARCO* (a class A scavenger receptor), *MSR1* (CD204), *FCGR1A* (CD64), and *CD163* (50). *CD163* was previously reported in macrophages in the setting of liver-inflammatory diseases (51, 52). However, these genes were not modulated in the lungs of SIV^hi^-infected RMs (Figure 5B).

In SIV^lo^-infected RMs, *CXCL10* and *CXCL11* were also increased in hepatic tissue associated with higher levels of *MSR1* (CD204), *CSF1R*, and *CD163*, whereas *FCGR1A* (CD64) was downregulated in the lungs (Figure 5B). Furthermore, our results demonstrated that these transcripts were downregulated in ART-treated RMs, compared with SIV-infected RMs, and no major difference was observed between ART-treated and healthy uninfected RMs (Figure 5B), except for *MPEG1* being downregulated in the lungs of ART-treated RMs. Thus, transcriptomic analyses revealed M1 macrophages in hepatic and pulmonary tissues in which only TRM markers were increased in the former, mostly in highly viremic RMs.

**Figure 6:**
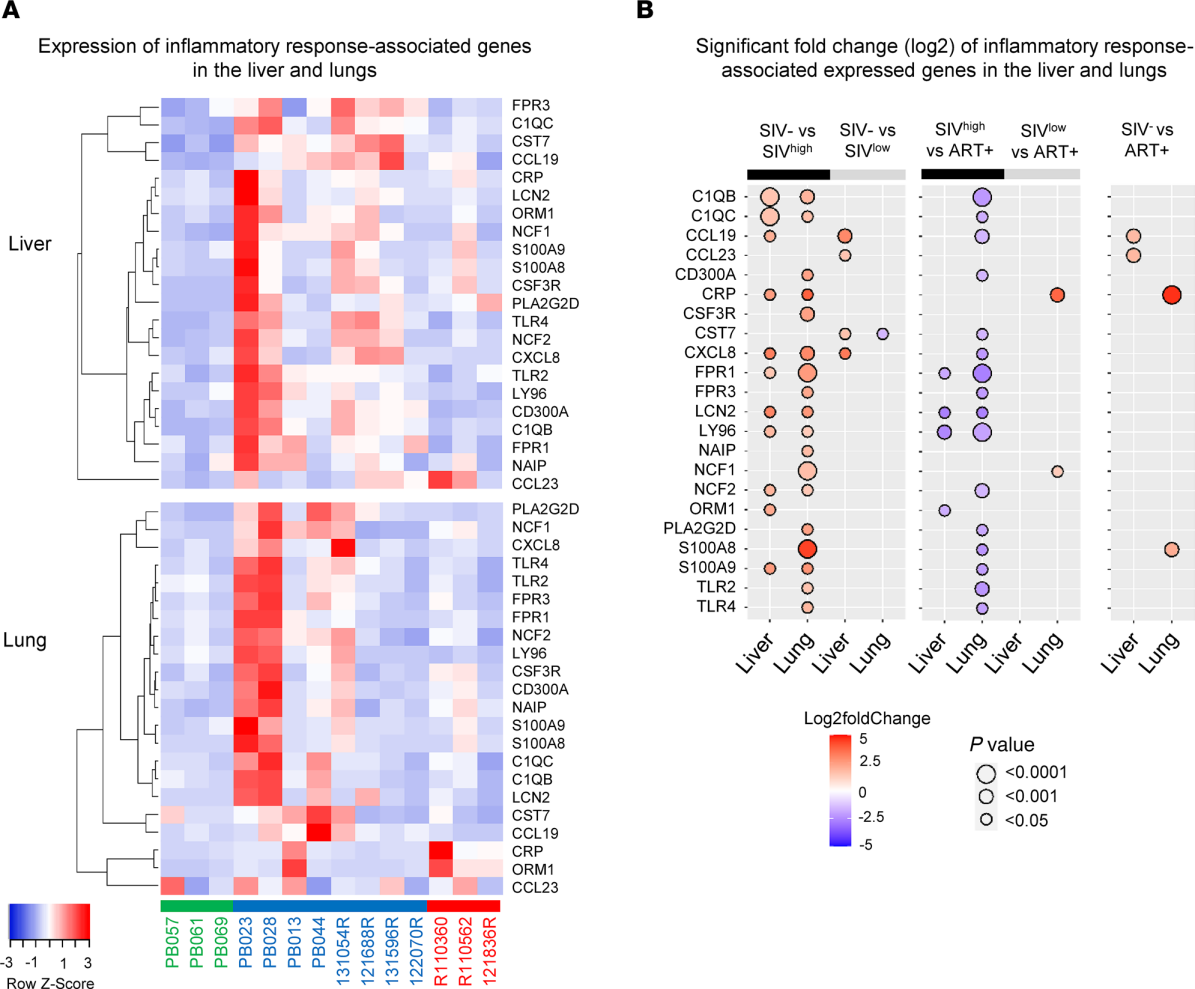
Expression of inflammatory response–associated genes in the liver and lungs of untreated SIV-infected and ART-treated RMs. (**A**) Heatmaps show the differential expression of inflammatory response–associated genes in the liver and lungs between naive RMs (*n* = 3), SIV-infected RMs (*n* = 8), and ART-treated RMs (*n* = 3). Row *z* score shows the differential expression of a single gene across the samples. Red bars indicate an increased abundance of the corresponding genes, and blue bars indicate a decreased abundance. (**B**) Significant log_2_ fold-change of inflammatory response transcripts between i) naive and SIV-infected RMs (left panel), ii) SIV-infected and ART-treated RMs (middle panel), and iii) naive and ART-treated RMs (right panel). The bidirectional color-coded bubbles represent the log_2_ fold-change *z* score, whereas the size of the bubbles indicates the –log_10_ (*P* value) with a threshold of *P* < 0.0001 for highly significant values. SIV^hi^, highly viremic SIV; SIV^lo^, low viremic SIV.

Consistent with higher levels of M1 macrophage transcripts, we also observed higher levels of those related to inflammation (Figure 6A). The significant log_2_ fold-changes, associated with up- and downregulated genes, are shown in Figure 6B and Supplemental Table 2, A and B. Thus, in SIV^hi^-infected RMs, we found higher levels of *CXCL8*, complement proteins (*C1QB*, *C1QC*), *CRP*, and neutrophil transcripts (*NCF2*, *S100A9*, *FPR1*, *LCN2*) both in hepatic and in pulmonary tissues (Figure 6B). We also observed additional inflammatory transcripts in the lungs of SIV^hi^-infected RMs compared with healthy uninfected RMs, including *S100A8*, *NCF1*, *FPR1*, *CSF3R*, and Toll-like receptor (*TLR2* and *TLR4*). In SIV^lo^-infected RMs, few genes were significantly modulated. We observed higher levels of *CXCL8*, *CCL19* (MIP-3β), and *CCL23* (MIP-3) in the liver only (Figure 6B). We also noticed that in ART-treated RMs, compared with healthy uninfected RMs, 2 genes, *CCL19* and *CCL23*, persisted in the liver, whereas *CRP* and *S100A8*, considered masters of the inflammatory signature, were increased in pulmonary tissue, despite early ART (Figure 6). In addition, the lungs of early ART-treated RMs displayed a discriminative signature corresponding to the acute-phase proteins (such as *AHSG*, *APCS*, *CRP*, *HAMP*, *LPB*, *PROC*, and *TFR2*), complement (*C6*, *C8A*, *C9*, and *CFHR5*), and coagulation (*FGA*, *FGB*, *FGG*, *FGL1*, *F2*, *F12*, *HP*, and *KNG1*) (Supplemental Table 2B) that was consistent with the observation of a persistent signature of tissue damage (Supplemental Figure 4).

Because injury and inflammation may be associated with altered cellular metabolic pathways, such as lipotoxic injury fueling inflammation by attracting innate cells (53), we analyzed associated transcripts. Transcriptomic analyses of SIV^hi^-infected RMs revealed genes related to lipid/steroid metabolic pathways (as shown in Supplemental Figures 5 and 6). Globally, in the liver, transcripts involved in the acetyl-CoA metabolic process were upregulated (*ACSS2*, *HMGCS1*, and *MSMO1*), supporting cholesterol and steroid synthesis (*HSD3B2*) (Supplemental Figure 4A), whereas *ME1* (NADP-dependent enzyme that generates NADPH for fatty acid) was downregulated (Supplemental Figure 5A). In addition, phospholipase A2 family transcripts (*PLA2G2A* and *PLA2G4C*), known for their contribution in the biosynthesis of inflammatory mediators, were increased. The *P* values and log_2_ fold-changes, associated with up- and downregulated genes, are presented in Supplemental Figure 6 and listed in Supplemental Table 2, A and B.

In the lungs, we also observed an altered metabolic pathway in which the number of transcripts was higher compared with the liver and associated with steroid glucuronidation (*UGT1A1/UGT2B7*), foam cell formation (*PON1*), and PPARα pathway (*FABP1*, *APOA5*, *UGT1A1*, *HMGCS2*) (Supplemental Figure 5B and Supplemental Figure 6A). Members of the phospholipase A2 family were also increased. Metabolism disturbance is also marked by the upregulation of cysteine, serine, and S-adenosyl methionine synthesis–associated genes and by the upregulation of tryptophan, histidine, phenylalanine, and tyrosine catabolism (Supplemental Figure 5B and Supplemental Figure 6A).

Interestingly, we observed that several expressed genes remained elevated in early ART-treated RMs, associated with lipid metabolism (*APOA1*, *APOA5*, *APOB*, *APOC2*, *APOD*, *APOF*, *PON1*, and *FABP1*), retinol (*ADH4*, *HSD11B1*, *HSD17B6*, *RDH16*, *RBP4/5*, and *TTR*), steroid (*CYP2E1*, *CYP3A7*, *GNMT*, *UGT1A1*, and *UGT2B7*), pyruvate (*AGXT*, *HPD*, *PCK1*), and tryptophan pathways (*PAH*), as well as several methyl-acyl-glucuronosyltransferases (*GLYAT*, *GNMT*, *MAT1A*, *TAT*, *UGT1A1*, and *UGT2B7*) (Supplemental Figure 6). Thus, although early ART drastically reduced the extent of the innate immune response in the liver and lungs of SIV-infected RMs, a persistent alteration of cell metabolism was observed in pulmonary tissues.

Together, our results support higher M1 macrophage gene profiles associated with an inflammatory environment profile in SIV^hi^-infected RMs (54) in which altered metabolic pathways persisted in lungs, despite early ART.

### CD183 and CX3CR1 are expressed by TRM-like cells in the inflamed liver and lungs of SIV-infected RMs.

**Figure 7:**
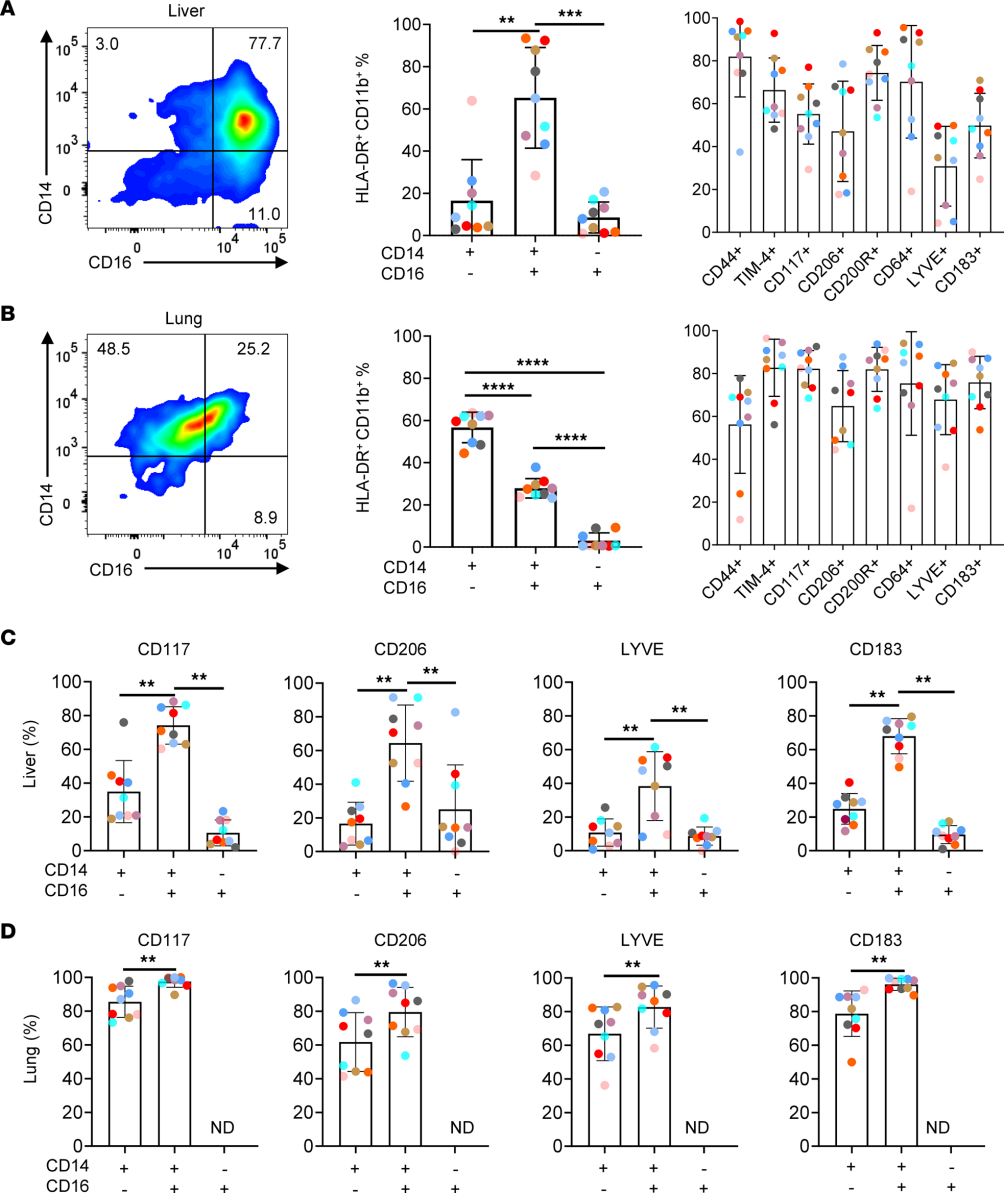
Phenotypes of HLA-DR^+^CD11b^+^ myeloid cells in the liver and lungs of untreated SIV-infected RMs. Left, representative dot plots depicting the expression of CD14 and CD16 among CD3^–^CD20^–^HLA-DR^+^CD11b^+^ cells. The percentages of classical (CD14^+^CD16^–^), intermediate (CD14^+^CD16^+^), and nonclassical (CD14^–^CD16^+^) cells and TRM markers are indicated in the (**A**) liver and (**B**) lungs. Histograms show the percentages of TRM markers in classical, intermediate, and nonclassical subsets in the (**C**) liver and (**D**) lungs. Each colored symbol represents 1 individual (*n* = 9). A 1-tailed Wilcoxon’s test was performed; ** *P* < 0.01, *** *P* < 0.001, **** *P* < 0.0001. Data represent mean ± SD. ND, not determined.

To better understand the heterogeneity of pulmonary and hepatic myeloid cell populations in SIV-infected RMs, we stained cells with a cocktail of antibodies and analyzed by flow cytometry. First, we excluded CD3^+^ and CD20^+^ cells, and myeloid cells were gated on HLA-DR^+^ and CD11b^+^ (excluding neutrophils, which are HLA-DR^–^). Initially, the expressions of CD14 and CD16 were analyzed. The percentages of CD14^+^CD16^+^ cells were 65.2% ± 24% in the liver of SIV-infected RMs (Figure 7A) while most of the cells in the lungs were CD14^+^CD16^–^ (61.0% ± 11%) (Figure 7B). As shown, these subsets of CD14 and CD16 population also expressed CD45, excluding the role of CD45^–^ cell populations (hepatocytes and endothelial cells) (50) (Supplemental Figure 7). Because TRMs in RM are not well defined compared with the mouse model, we analyzed the expression of 8 TRM markers in SIV-infected RMs. Our results indicated that CD11b^+^ cells from the lungs and liver of SIV-infected RMs (Figure 7, A and B) expressed high levels of CD117, CD206, and LYVE compared with CD11b^+^ cells from the spleen (Supplemental Figure 8A). However, whereas in mouse models CD44 and CD64 expressions have been proposed to be specific markers of TRMs, our results showed that these markers were also highly expressed on blood monocytes from SIV-infected RMs (Supplemental Figure 8B). Therefore, CD206, CD117, and LYVE were the most specific markers. Thus, the percentages of CD11b^+^ expressing CD206 were 64.8% ± 17% in the lungs and 47.1% ± 23% in the liver (Figure 7, A and B), whereas only 6.0% ± 5% were observed in the spleen (Supplemental Figure 8A). Those expressing CD117 were 55.1% ± 14% in the lungs and 82.2% ± 8% in the liver and only 9.2% ± 10% in the spleen. The percentages of CD11b^+^LYVE^+^ cells were 67.8% ± 16% in the lungs and 30.8% ± 18% in the liver, whereas only 5.2% ± 4% were in the spleen. To exclude a possible contamination of circulating blood monocytes in the liver and lungs, blood cell profiles were also examined, demonstrating the absence of CD206 expression both in CD14^+^ and CD16^+^ cells (Supplemental Figure 4C). Similarly, neither CD117 nor LYVE was expressed in blood monocytes (Supplemental Figure 8B). The specificity of PE and APC stainings (fluorescence minus one) is shown (Supplemental Figure 9). As a comparison, we also showed staining from CD11b^+^ (Supplemental Figure 10A) versus CD11b^–^ cells (Supplemental Figure 10B).

We then analyzed CD206, CD117, and LYVE expressions on the surface of CD14 and CD16 cell subsets. Interestingly, the inflammatory CD14^+^CD16^+^ cell subset expressed the highest levels of TRM markers compared with the CD14^+^CD16^–^ and CD14^–^CD16^+^ cell subsets (Figure 7, C and D). Indeed, recent studies also observed the acquirement of TRM markers by monocyte-derived macrophages infiltrating inflamed tissues (32–34, 55). This observation raises the question of recruitment of these cells. Transcriptomic analyses revealed a higher level of CXCL10 (IP-10) gene transcript in SIV-infected RMs. Therefore, we assessed the expression of CD183 (CXCR3), the receptor of CXCL10/IP-10. Thus, the percentages of CD11b^+^ cells expressing CD183 were 75.8% ± 12% in the lungs and 49.8% ± 15% in the liver (Figure 7, A and B). CD183 was expressed on CD14^+^CD16^+^ cells (Figure 7, C and D) and CD11b^+^LYVE^+^ cells from liver and lungs of SIV-infected RMs (Supplemental Figure 10A). As a control CD11b^–^ populations are shown (Supplemental Figure 10B). Among the other factors, the fractalkine receptor, CX3CR1, expressed by blood CD16^+^ cells (7, 56) (Supplemental Figure 11), has been reported to participate in the recruitment of inflammatory monocytes across human liver sinusoidal endothelium (57), in inflamed and damaged tissues (13, 55), and to control their survival (58, 59). Herein, we found that CX3CR1 was expressed by CD14^+^ and CD16^+^ cell subsets (Supplemental Figure 11) both in the lungs and liver of SIV-infected RMs.

Together, our results point out the role of monocyte-derived macrophages infiltrating inflamed tissues associated with the expression of CD183 and CX3CR1, giving rise to TRM-like macrophages in the context of inflammation and tissue damage in SIV-infected RMs (60–62).

### Viral infection of macrophage subsets in the liver and lungs of SIV-infected RMs.

**Figure 8:**
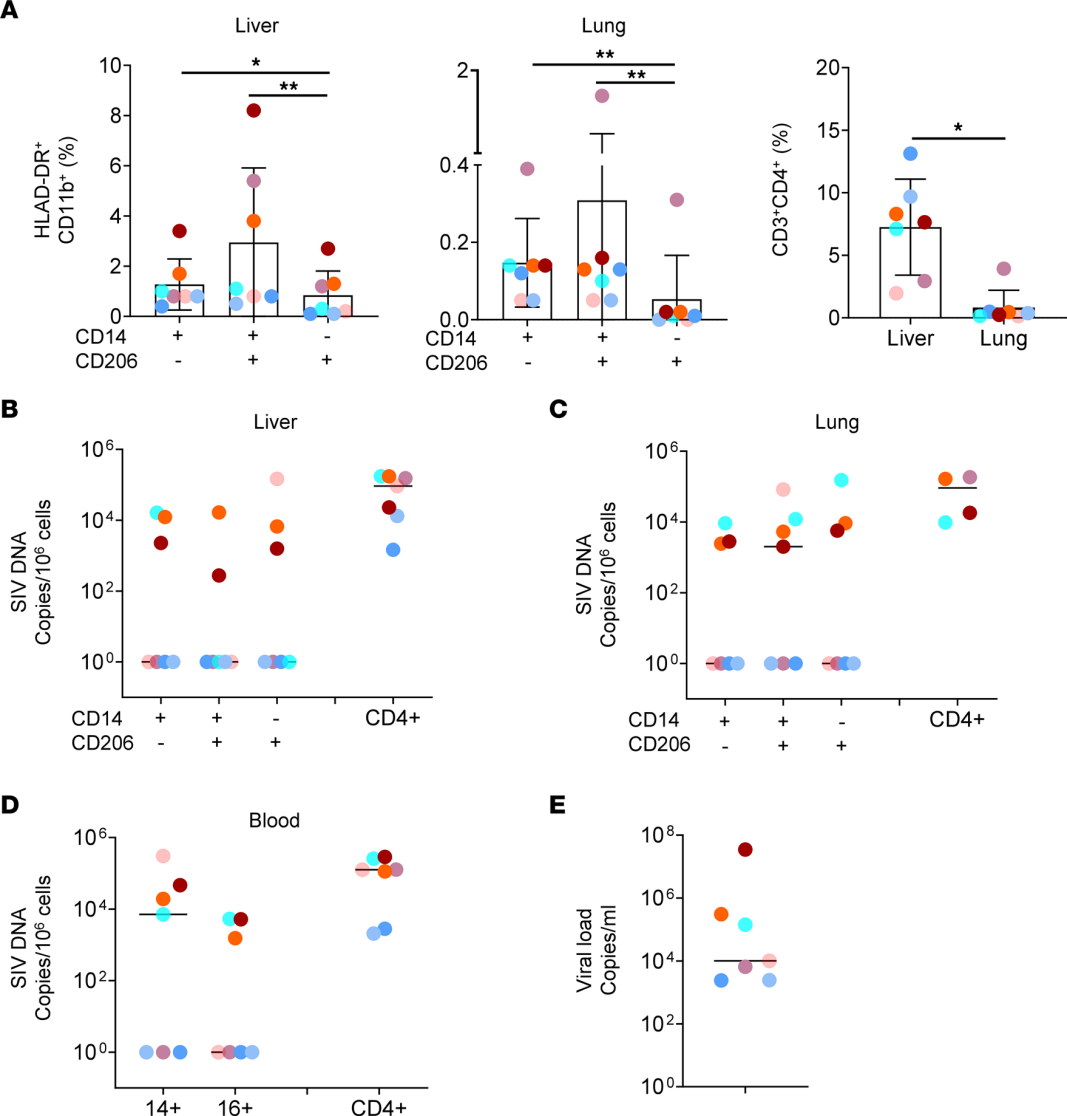
Frequencies of cell-associated SIV DNA of macrophage subsets in the liver and lungs of untreated SIV-infected RMs. (**A**) Histograms show the absolute percentages of sorted myeloid subsets and CD4^+^ T cells. Data represent mean ± SD. Frequencies of cell-associated SIV DNA were quantified by qRT-PCR in sorted macrophage subsets and in CD4^+^ T cells from the (**B**) liver and (**C**) lungs (*n* = 7). In the lungs, we only tested 4 individuals for CD4^+^ T cells, due to the low number of cells. (**D**) SIV DNA was quantified in sorted monocyte subsets (CD14^+^ and CD16^+^) and in CD4^+^ T cells from the blood for the same animals. Results are expressed as copies per 10^6^ cells. (**E**) Viral loads in the plasma were quantified for each RM; results are expressed as viral load copies per milliliter. Bars indicate median values. Each color represents 1 individual. A 1-tailed Wilcoxon’s test was performed; * *P* < 0.05, ** *P* < 0.01.

Given the diversity of macrophage populations, we decided to analyze the nature of infected cells in the liver and lungs of SIV-infected RMs. We sorted CD14^+^CD206^–^, CD14^+^CD206^+^, and CD14^–^CD206^+^ cell subsets from CD11b^+^HLA-DR^+^ cells that may reflect the different subsets from infiltrating monocytes to transient inflammatory macrophages to TRMs. Cell gating strategy and purity are shown in Supplemental Figure 12. We showed in Figure 8A the percentages of sorted macrophage cells in the liver and lungs. The percentages of CD4^+^ T cells are also shown in Figure 8A. In 4 RMs in which viral DNA was detected (Figure 8, B and C) in the 3 cell subsets (CD14^+^206^–^, CD14^+^206^+^, and CD14^–^206^+^), we also detected viral DNA in blood monocytes (Figure 8D). These RMs displayed the highest viremia (higher than 10^4^ copies/mL) (Figure 8E). On the contrary, none of these cell subsets contained viral DNA in the liver and lungs of SIVmac251-infected RMs (Figure 8, B and C) in which viral DNA in blood monocytes was undetectable (Figure 8D) and displayed low viremia (lower than 10^4^ copies/mL) (Figure 8E). Due to the number of sorted cell subsets, viral RNA was not quantified. In addition to myeloid cells, we quantified the levels of viral DNA in CD4^+^ T cells. These levels were 7.6 × 10^4^ copies/10^6^ cells in the liver and 1.4 × 10^5^ copies/10^6^ cells in the lungs (Figure 8, B and C). In the lungs, only 4 RMs were analyzed, due to limited numbers of cells recovered from SIVmac251-infected RMs. Therefore, the levels of viral DNA were 10-fold higher in CD4^+^ T cells than what was observed in myeloid cells.

**Figure 9:**
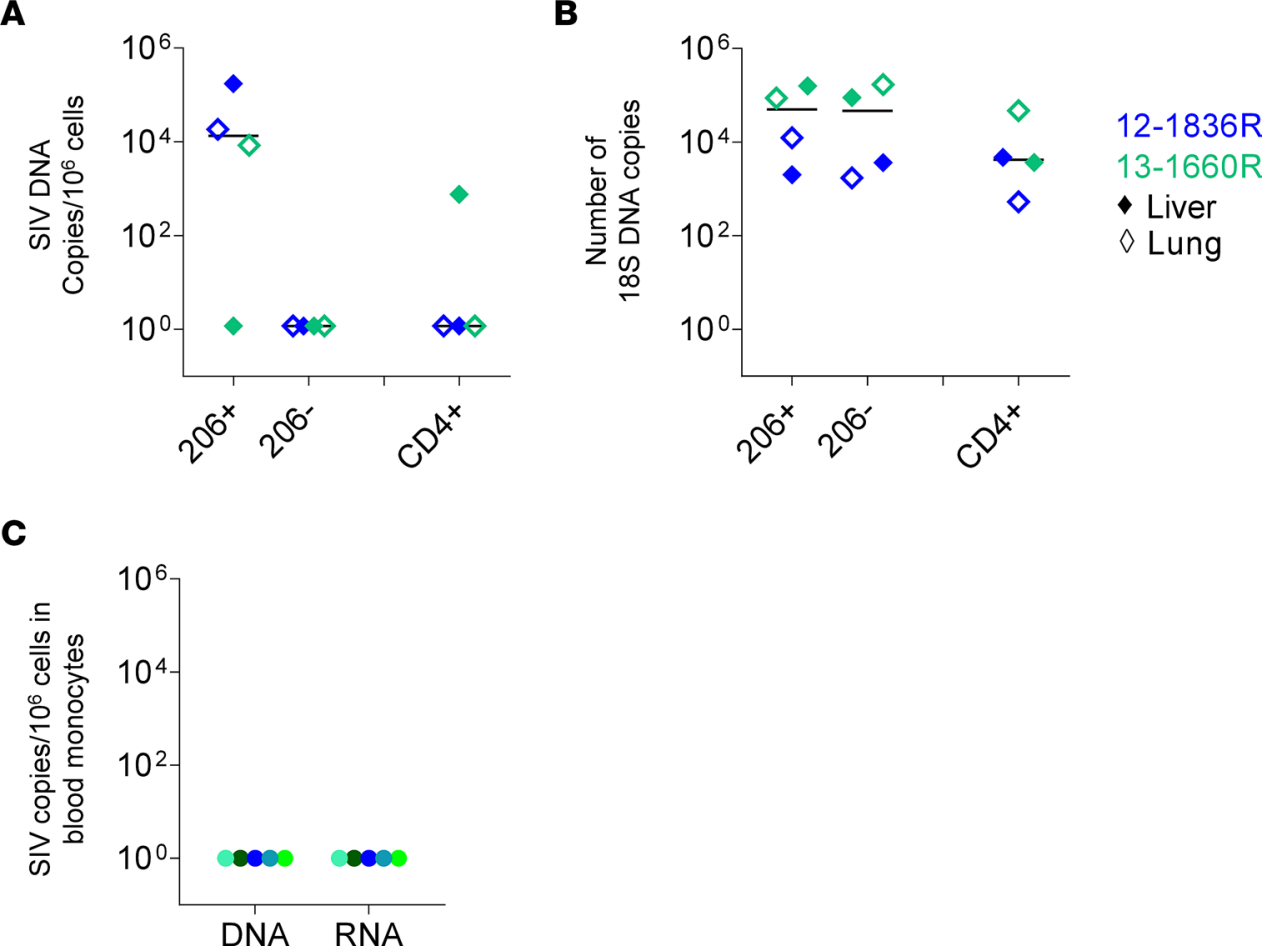
Frequencies of cell-associated SIV DNA of macrophage subsets in the liver and lungs of ART-treated RMs. (**A**) Frequencies of cell-associated SIV DNA and (**B**) number of 18S DNA copies were quantified by qRT-PCR in sorted CD206^+^ and CD206^–^ subsets of the liver and lungs in ART-treated RMs (*n* = 2). Results are expressed as copies per 10^6^ cells. Bars indicate median values. Each color represents 1 individual. (**C**) Frequencies of SIV DNA and RNA were quantified by qRT-PCR in sorted CD14^+^ blood monocytes of ART-treated RMs (*n* = 5). Filled circle symbols, blood samples; filled diamond symbols, liver samples; unfilled diamond symbols, lung samples.

We then assessed the extent of infected cells in SIV-infected RMs having initiated ART on day 4 postinfection. We sorted CD11b^+^HLA-DR^+^ expressing or not CD206. Indeed, due to the low number of cells recovered from ART SIV-infected RMs, it was not possible to analyze more subsets. In parallel, CD4^+^ T cells were sorted from the same individuals. Our results indicated that viral DNA was detected in CD206^+^ myeloid cells and not in CD206^–^ cell subset (Figure 9A). The absence of viral DNA detection was not related to a difference in the quantity of cells analyzed, as indicated by the expression of 18S DNA (Figure 9B). In the blood, we did not detect viral DNA or RNA in sorted monocytes (Figure 9C). Assessing CD4^+^ T cell infection, we found that viral DNA was detected only from the liver of RM 13-1660R, whereas we did not detect viral DNA in CD206^+^ cells in the liver of this animal. Thus, under ART, the pool of infected cells was extremely low.

We then analyzed the extent of viral seeding in the lungs and liver of RMs after ATI (Supplemental Figure 13A). Thus, we analyzed RMs, in which we previously demonstrated a viral rebound after ATI (Supplemental Figure 13, A and B) (3). RMs were sacrificed at day 12 (121888R), 15 (R110804 and 131134R), 18 (111430R), and 28 (131878R), 3 days after the first detection of a VL after ATI. In RMs in which we detected viral DNA/RNA in blood monocytes (Supplemental Figure 13C), although we did not perform the viral quantification in sorted cells in the liver and lungs, we also detected viral DNA/RNA in both tissues (Supplemental Figure 13, D and E). SIV DNA reached 6.9 × 10^3^ copies/10^6^ cells in the lungs and 1 × 10^4^ copies/10^6^ cells in the liver. The levels of SIV RNA were 3.4 × 10^5^ copies/10^6^ cells in the lungs and 3.3 × 10^3^ copies/10^6^ cells in the liver. Conversely, animals in which we did not detect viral DNA/RNA in blood monocytes (Supplemental Figure 13C) had no measurable DNA and RNA in the lungs and liver (Supplemental Figure 13, D and E). Thus, viral dissemination in the lungs and liver is extremely rapid and is observed less than 2 weeks after ATI.

Together, these observations indicate that early ART reduces viral seeding in both the liver and lungs.

## Discussion

In this study, we demonstrated that infection in the liver and lungs of SIV-infected RMs is associated with the innate immune response, reflecting the sensing of SIV, which is prevented by the administration of early ART. Transcriptomic analysis also highlighted the complexity of immune cells recruited in inflamed and damaged tissues, as indicated by the levels of gene transcripts related to inflammasome and pyroptosis (35, 45). This complexity in macrophage populations, in the context of viral infections, is highlighted by the observation that CD14^+^CD16^+^ cells also expressed CD206 and LYVE, which characterized TRM cells, as well as expressing CD183 and CX3CR1. In the context of an increased expression of mRNA coding for chemokines, these results support the recruitment of monocyte-derived macrophages in inflamed tissues. Our results also showed that only in the highest viremic RMs, viral DNA is detected in CD14^–^206^+^, CD14^+^CD206^–^, and CD14^+^CD206^+^ hepatic and lung cell populations. Together, our results support the interest of early ART to drastically reduce viral dissemination and inflammation in these tissues, which have been associated with several comorbidities in HIV-infected patients (63–65).

Macrophages are heterogeneous cell populations involved in tissue homeostasis (66). Whereas long-lived macrophages have been described in newborn mice (17, 18), after extravasation in adults, monocytes may differentiate into macrophages related to environmental conditions, such as in the context of tissue damage, microbial infections, and inflammation (56). Furthermore, recent findings in human inflammatory diseases have challenged the importance of these markers to define TRM cells in which macrophages once recruited at the inflammatory sites may also acquire these markers (31–34). Thus, once differentiated into long-lived resident macrophages, they persist longer, replacing those that have been depleted (67). In addition to transcriptomic analyses that demonstrate the heterogeneity of myeloid cell populations, flow cytometry revealed that most of the macrophages display a CD14^+^CD16^+^ phenotype. In chronically inflamed human liver tissue, Liaskou et al. (61) reported an accumulation of CD14^+^CD16^+^ cells as a consequence of enhanced recruitment from blood and local differentiation. Our results also indicated that CD11b^+^CD14^+^CD16^+^ cells also express CD117, LYVE, and CD206, both in the lungs and liver of SIV-infected RMs. This complexity of macrophage populations is also supported by other reports (33, 34, 68). Thus, Tan-Garcia et al. showed an enrichment of CD14^+^HLA-DR^hi^CD206^+^ myeloid cells in the context of viral related liver disease, contributing to inflammation (60). The plasticity of macrophage populations is also challenged by other studies showing the role of the CSF1 in the recruitment and the differentiation of inflamed macrophages into resident macrophages, after tissue injury (19, 20).

Furthermore, the expression of CD183 (CXCR3) and CX3CR1 by CD11b^+^CD14^+^CD16^+^ cells strongly suggested that monocyte-derived macrophages infiltrate the inflamed tissues. Although little attention has been given to the expression of CD183, Zhou et al. (34) have reported that CD183 is important for perivascular macrophage accumulation and arterial remodeling. Thus, the observation of higher levels of CXCL10 in the lungs and liver of SIV-infected RMs may lead to the recruitment of myeloid cells expressing CD183. This is consistent with previous reports showing portal and perivenular cell infiltration in the liver, as well as infiltrated cells in the lungs of SIV-infected RMs (40, 41, 69, 70). Of importance, our results highlighted that early ART prevents not only viral seeding but also CXCL10 mRNA expression that can limit the recruitment of myeloid cells fueling local inflammation. Whereas CX3CR1 is normally lost during the development of TRMs in mice (71), we have also observed that CX3CR1 is expressed by CD11b^+^CD14^+^CD16^+^ cells. Dal-Secco et al. reported that monocytes can develop into resident CX3CR1 macrophages in the liver at the site of a sterile injury (13). In the gut, CCR2^+^ blood monocytes can give rise to macrophage subsets in an inflamed colon, leading to resident CX3CR1^hi^ macrophages (72–75) in which CX3CR1/CX3CL1 axis is involved in the survival of resident macrophages (76–78).

In this context, we observed that myeloid cell subsets, including CD11b^+^CD14^–^206^+^, CD11b^+^CD14^+^206^+^, and CD11b^+^CD14^+^206^–^, are infected. However, it is noteworthy that we detected viral DNA only in RMs in which viremia was the highest (>10^4^ copies/mL) and a frequency higher than 10^2^ SIV DNA copies per million of blood monocytes. Of importance, we noticed that viral DNA was only detected in CD206^+^ cells after early ART. Because of the limited quantity of materials, we cannot exclude that viral DNA detected in macrophages reflects the phagocytosis of dying CD4^+^ T cells, as previously reported in the liver (43). The high levels of TIM-4 on the cell surface of macrophages from both the lungs and liver may support a role in the clearance of dead cells (24, 25, 79), which have been correlated with AIDS disease severity (80–84). Thus, in addition to macrophages, we found that CD4^+^ T cells are infected in the liver and lungs. The frequencies of infected cells were at least 10-fold higher in CD4^+^ T cells than in macrophages.

Whereas infectious virus has been reported to persist in macrophages from the lungs in ART-treated chronically SIV-infected pigtailed monkeys (85) or in ART-treated HIV-infected adults (86), other groups have noted the absence of viral DNA in ART-treated HIV^+^ patients (87) or in bronchoalveolar lavage of ART-treated SIV-infected RMs (88). In the liver, macrophages of ART-treated SIV-infected RMs displayed low viral DNA (40) and were reported to be defective to propagate HIV in people living with HIV (PLWH) (89). Herein, the impact of early ART in drastically reducing viral DNA in the liver and lungs, concomitant with the absence of viral detection in monocytes subsets, suggest a positive impact of peripheral blood monocytes in the viral dissemination in these tissues. This low level of viral detection under early ART is consistent with transcriptomic analysis, indicating that, in addition to the inflammatory signature, the gene transcripts of ISGs (ISG15/ISG20 and IFIT/IRF/TRIM family) related to viral sensing (44, 90) are drastically reduced after early ART administration. This observation is also important because genes related to inflammasome, pyroptosis (35), and lysoptosis (45) are also reduced in early ART-treated RMs. This is of interest for PLWH, to limit viral dissemination in the lungs and liver, thus reducing comorbidities associated with liver and lung tissue damage (63–65, 91, 92).

Our results also indicated that metabolic pathways including glycolysis, steroid synthesis, lipid, and tryptophan catabolism are altered in SIVmac251-infected RMs. Given the heterogeneous macrophage cell fates, they can contribute to sustaining inflammation, steatosis, and fibrosis (93, 94). However, despite early ART, our results indicated that, contrary to the liver, several gene transcripts associated with the acute-phase response, coagulation, and complement remain elevated in the lungs. This persistent alteration of gene expression could contribute to the tissue damage observed in PLWH (95). Serpins are important regulators of extracellular matrix remodeling, contributing to lung pathology, such as COPD and cystic fibrosis (46), while tissue factor drives coagulopathy (96). We also observed genes associated with metabolic pathways, such as indoleamine 2,3-dioxygenase (IDO), that regulate tryptophan catabolism. IDO was previously shown to be associated with an altered Th17/Treg balance in PLWH (97) and abortive CD8^+^ T cell survival in SIV-infected RMs (98). The presence of CD206-infected cells in the tissues despite ART could contribute to altering the metabolism. This may also provide support to the observation that several genes corresponding to the extracellular matrix organization, acute-phase proteins, complement, coagulation, and lipid metabolism remain elevated in the lungs.

This current study has potential limitations. Given that we prioritized viral DNA quantification for measuring viral reservoirs because of limited number of sorted cells from liver and lung tissues, we did not assess phagocytosis issues by quantifying *CD3E* transcripts in macrophages, as we performed previously for monocytes (3). Furthermore, although the phenotype of pulmonary/hepatic cells is different from that observed in the blood, we cannot exclude blood contamination in our analyses. Performing a CD45 intravascular staining could be of interest to separate contaminating peripheral cells from tissue-resident cells. Finally, the limited number of ART-treated animals analyzed for measuring viral reservoir is also to be considered.

Overall, our data highlight the presence of CD16^+^CD14^+^CD206^+^LYVE^+^ cells in the lungs and liver of SIV-infected RMs. Thus, a pro-inflammatory environment in both tissues strongly favors the recruitment of inflammatory monocytes (CD14^+^CD16^+^) that subsequently acquire resident macrophage phenotypes. Of interest, we demonstrated that this macrophage population expresses CD183 and CX3CR1. Finally, our findings also provide evidence that early ART administration efficiently inhibits inflammation, as well as viral seeding, in hepatic and pulmonary tissues. Therefore, it is of crucial importance to limit this vicious cycle of viral infection of myeloid cells in the lungs and liver early, reducing the comorbidities associated with liver and lung tissue damage.

## Methods

### Animals, viral inoculation, and sample collection.

Twenty-five RMs seronegative for SIV, simian T leukemia virus type 1, SRV-1 (type D retrovirus) and herpes B virus were infected intravenously with SIVmac251 (20 AID_50_). At day 4 postinfection, 10 RMs were treated with tenofovir (20 mg/kg, Gilead) and emtricitabine (40 mg/kg, Gilead) subcutaneously and raltegravir (20 mg/kg, Merck) or dolutegravir (5 mg/kg, ViiV) and ritonavir (20 mg/kg; AbbVie) by oral route. Five individuals of the early ART-treated RMs interrupted the treatment 8 weeks after ART administration. RMs were sacrificed at different time points postinfection during natural infection (no ART, *n* = 15), under ART (ART, *n* = 5), and after ATI (*n* = 5), as shown in Supplemental Table 1. Liver, lungs, and spleen were recovered immediately after euthanasia for flow cytometry analysis and cell sorting. Cells were isolated after a mechanical process (3). To limit the negative effects on the expression of cell surface markers, tissues were not digested with collagenase or other proteases.

### Immunophenotyping.

After a mechanical process, fresh cells from the liver, lungs, and spleen were resuspended in RPMI medium (Thermo Fisher Scientific) and stained with monoclonal antibodies (Supplemental Table 4A) in the dark for 30 minutes at 4°C. Cells were washed in PBS twice by centrifugation at 300*g* for 7 minutes. After lysing erythrocytes (Lysing buffer Pharm Lyse 10×, BD Biosciences) for 15 minutes, cells were washed and resuspended in cold paraformaldehyde 1%. Then, 60,000 events corresponding to mononuclear cells were recorded in FACSCelesta (BD Biosciences). Analyses were performed using FlowJo software (Tree Star).

### RNA sequencing.

RNA from liver and lung cell samples in TRIzol (Thermo Fisher Scientific) were extracted using QIAGEN RNeasy Micro Kit with DNase I treatment. Quantity and quality of total RNA were assessed by ND-1000 spectrophotometer (NanoDrop Technologies) and Bioanalyzer 2100 (Agilent RNA 6000 Pico Kit). The NEBNext Ultra II directional RNA library prep kit for Illumina (New England Biolabs) was used to prepare libraries, according to the manufacturer’s instructions. Briefly, 400 ng of total RNA was purified using the NEBNext poly(A) and used as a template for cDNA synthesis by reverse transcriptase with random primers. The specificity of the strand was obtained by replacing the dTTP with the dUTP. This cDNA was converted to double-stranded DNA that was end-repaired. Ligation of adaptors was followed by a purification step with AxyPrep Mag PCR Clean-up kit (Axygen), by an excision of the strands containing the dUTPs, and finally, by a PCR enrichment step of 9 cycles to incorporate specific indexed adapters for the multiplexing. The quality of final amplified libraries was examined with a DNA screentape D1000 on a TapeStation 2200, and the quantification was done on the QuBit 3.0 fluorometer (Thermo Fisher Scientific). mRNA-Seq libraries with unique index were pooled together in equimolar ratio and sequenced for paired-end 100 bp sequencing on an NovaSeq 6000 flowcell S1 at the Next-Generation Sequencing Platform (CHU de Québec Research Center). The average insert size for the libraries was 290 bp. The mean coverage/sample was 24 million paired-end reads.

### Transcriptome analysis.

Reads were trimmed using fastp v0.20.0, and the quality of the reads was assessed using FastQC v0.11.8 and MultiQC v1.8. The quantification was performed with Kallisto v0.46.2 against the *Macaca mulatta* transcriptome (Mmul_10 downloaded from Ensembl release 103). The PCA was completed with the FactoMineR v2.4 R package. The PCA and volcano graphical representations were produced with the ggplot2 v3.3.3 package. Differential expression analysis was performed using the DESeq2 v1.30.1 package. This package overcomes the lack of power, due to the high uncertainty of the variance estimation, by pooling information across genes (99). Differentially expressed genes were defined as genes with an adjusted *P* value smaller or equal to 0.05, a fold-change greater than 2.5 or smaller than 0.4, and a mean transcripts per million count across all samples greater than or equal to 2. All R analysis was done with R v4.0.3. Gene Ontology Biological Process terms (GO_BP) were analyzed using the ToppGene (ToppFun) tool (100). Gene classifications in Supplemental Table 2, C and D, were performed by combining 3 web interfaces: g:Profiler (101), GeneCodis 4.0 (102), and ToppGene (100). Genes related to metabolism pathways were classified by using the Reactome and KEGG Knowledgebase interface (103, 104).

### Cell sorting.

Liver and lung myeloid and CD4^+^ T cells were sorted using a BD Influx cell sorter upon staining with specific antibodies: anti-CD3, -CD4, -CD20, –HLA-DR, -CD11b, -CD14, and -CD206 mAbs (Supplemental Table 4A). Myeloid cells were identified as cells with a CD3^–^CD20^–^CD11b^+^HLA-DR^+^ phenotype and were sorted based on their differential expression of CD14 and CD206 into 3 different subsets, as follows: CD14^+^CD206^–^, CD14^+^CD206^+^, and CD14^–^CD206^+^. CD3^+^CD4^+^ T cells were sorted in parallel as controls. We used a cell-sorting strategy privileging purity (“pure mode”) versus enrichment, leading to a high level of purity of over 95% (Supplemental Figure 12). A minimum of 10^5^ cells were sorted per cell subset and preserved as dry pellets at –80°C for DNA quantification.

### Viral RNA quantification.

Plasmatic VLs were quantified by RT-qPCR using a PureLink Viral RNA/DNA Kit (Invitrogen). The PCR mixture was composed of 4× TaqMan Fast Virus 1-Step Master Mix (Applied Biosystems), 750 nM of primers, and 200 nM of probe. Primers and probe sequences are listed in Supplemental Table 4B. A plasmid coding for gag gene of SIVmac251 was used as a standard. Serial 10-fold dilutions of SIVmac251 plasmid were performed to generate a standard curve, starting at 10^9^ copies/μL of SIV. Amplifications were carried out with a QuantStudio 6 Flex Real-Time PCR System (Applied Biosystems). Samples were run in duplicate, and results are expressed as SIV RNA copies/mL (90).

### Cell-associated viral RNA quantification.

Viral RNA was assessed from 10^5^ cells. RNA was extracted with TRIzol procedure and resuspended in 50 μL of RNase-free water. DNA was eliminated using a TURBO DNA-free Kit (Invitrogen). We amplified 5 μL of RNA by qRT-PCR with a QuantStudio 6 Flex Real-Time PCR System (Applied Biosystems). Eukaryotic 18S rRNA Endogenous Control mix (Applied Biosystems) was used to estimate cell numbers in each sample. Primers and probe sequences are listed in Supplemental Table 4B. Samples were run in duplicate, and results are expressed as number of SIV RNA copies per 10^6^ cells.

### Cell-associated viral DNA quantification.

Viral DNA (from 10^5^ to 10^6^ cells) was purified using the Genomic DNA Tissue Kit (Macherey-Nagel). After elution (50 μL), 10 μL was amplified by nested PCR with SIVmac251-specific primers surrounding the nef coding region (105). A first round of PCR was performed using 50 nM of preco and K3 primers, 1.25 U of AmpliTaq (Applied Biosystems), and 0.8 mM DNTP (Invitrogen) in a Biometra thermocycler. A total of 5 μL of PCR product was re-amplified using 250 nM of SIV DNA primers and probe (Supplemental Table 4B) and 2× PrimeTime Gene Expression Master Mix (IDT) in a QuantStudio 6 Flex Real-Time PCR System. Samples were run in quadruplicate, and results are expressed as number of SIV DNA copies per 10^6^ cells. For DNA quantitation, serial dilutions of a plasmid were performed to generate a standard curve.

### mRNA quantification by quantitative PCR.

RNAs, from the liver and lungs of SIV-infected RMs, were reverse-transcribed using the SensiFAST cDNA Synthesis Kit (Bioline), following the manufacturer’s instructions. Quantitative PCR was performed in 10 μL using 2× QuantiTect SYBR Green PCR kit (QIAGEN) and 250 nM specific primers in a QuantStudio 6 Flex Real-Time PCR System. The sequence of each primer is provided in Supplemental Table 4B. The 18S was used as the housekeeping gene. Relative fold-change in gene expression was determined by the comparative cycle threshold (Ct) method using the formula 2^-ΔΔCt^. For each gene quantified, we used the Ct values for the healthy uninfected RM samples to obtain the ΔΔCt values.

### Statistics.

Statistics were analyzed using GraphPad Prism 8 software. The nonparametric Mann-Whitney test and the 1-tailed Wilcoxon’s test were used for comparison, as indicated in the figures. *P* values less than 0.05 indicate a significant difference. Spearman’s analysis was used for correlations.

### Study approval.

RMs were housed at Laval University, in accordance with the rules and regulations of the Canadian Council on Animal Care (http://www.ccac.ca). The protocol was approved by the Laval University Animal Protection Committee (Project number 106004). Animals were fed a standard monkey chow diet, supplemented daily with fruit and vegetables and water ad libitum. Social enrichment was delivered and overseen by veterinary staff, and overall animal health was monitored daily. Animals were evaluated clinically and were humanely euthanized, using an overdose of barbiturates, according to the guidelines of the Veterinary Medical Association.

### Data availability.

All data are available in the supplemental tables and Supporting Data Values file. RNA-Seq data were deposited in the NCBI’s Gene Expression Omnibus (GEO) and are publicly available under GEO accession GSE234399.

## Author contributions

JAC, MAJ, PA, and JE conceived and designed the experiments. JAC, HR, GR, CS, OZA, and JE performed the experiments. JAC, HR, GR, SB, OZA, CS, CJB, and JE analyzed the data. JAC, MAJ, AD, PA, and JE wrote the paper.

## Supplementary Material

Supplemental data

Supplemental table 2

Supplemental table 3

Supplemental table 4

Supporting data values

## Version 1 07/24/2023

Electronic publication
