# Supplementary material for: Early ART reduces viral seeding and innate immunity in liver and lungs of SIV-infected macaques
Source: JCI Insight. 2023 Jul 24;8(14):e167856. doi: 10.1172/jci.insight.167856 (PMC10443800; doi:10.1172/jci.insight.167856)
Supplement: Supplemental data [file jciinsight-8-167856-s154.pdf]

	Monkeys	Day of death PI	Viral Load	CD4/CD8 ratio	CD4 T cells (count/mm^3)	Age (years)	Symbol color
SIV-	PB057	0	0.00E+00	2.6	2475	4	
	PB061	0	0.00E+00	2.6	2684	3	
	PB069	0	0.00E+00	2.4	2191	4	
SIV+	9051222	33	1.58E+06	0.5	636	6	
	12-2070R	77	7.55E+05	0.6	860	5	
	PB023	167	1.39E+08	0.4	254	3	
	PB028	194	1.61E+08	0.2	370	3	
	PB044	223	5.71E+07	0.7	756	3	
	PB013	237	3.57E+06	1.0	764	3	
	12-1758R	295	3.68E+04	0.5	602	6	
	13-1298R	307	5.14E+05	0.2	190	5	
	12-1920R	310	1.34E+02	0.6	579	6	
	13-1572R	315	2.95E+04	0.3	415	6	
	13-1054R	314	6.54E+03	0.3	533	6	
	12-1688R	317	1.01E+04	0.1	205	6	
	13-1596R	321	2.40E+03	1.2	2141	6	
	13-1610R	331	1.43E+05	0.9	684	6	
	13-2054R	335	2.45E+03	1.1	1535	6	
SIV+ ART+	111466R	14	Undetectable	0.8	1550	5	
	R110562	27	Undetectable	1.0	1314	5	
	R110360	35	Undetectable	1.2	734	5	
	13-1660R	36	Undetectable	1.2	861	4	
	12-1836R	55	Undetectable	0.7	922	5	
SIV+ ATI		Day of death post-ART					
	12-1888R	12	7.66E+05	1.5	1450	6	
	R110804	15	3.71E+03	0.9	899	5	
	13-1134R	15	3.49E+07	2.4	1010	5	
	11-1430R	18	3.08E+05	1.5	1274	5	
	13-1878R	28	1.48E+03	0.8	569	5	

Table S1. Virological and immunological parameters of RM. For each animal, viral load, ratio of CD4/CD8 T cells, and CD4 T cell numbers from peripheral blood on the day of sacrifice are indicated. The age of each animal on the day of infection is indicated. The animals' symbol color used throughout the manuscript is shown. PI: Post-infection.

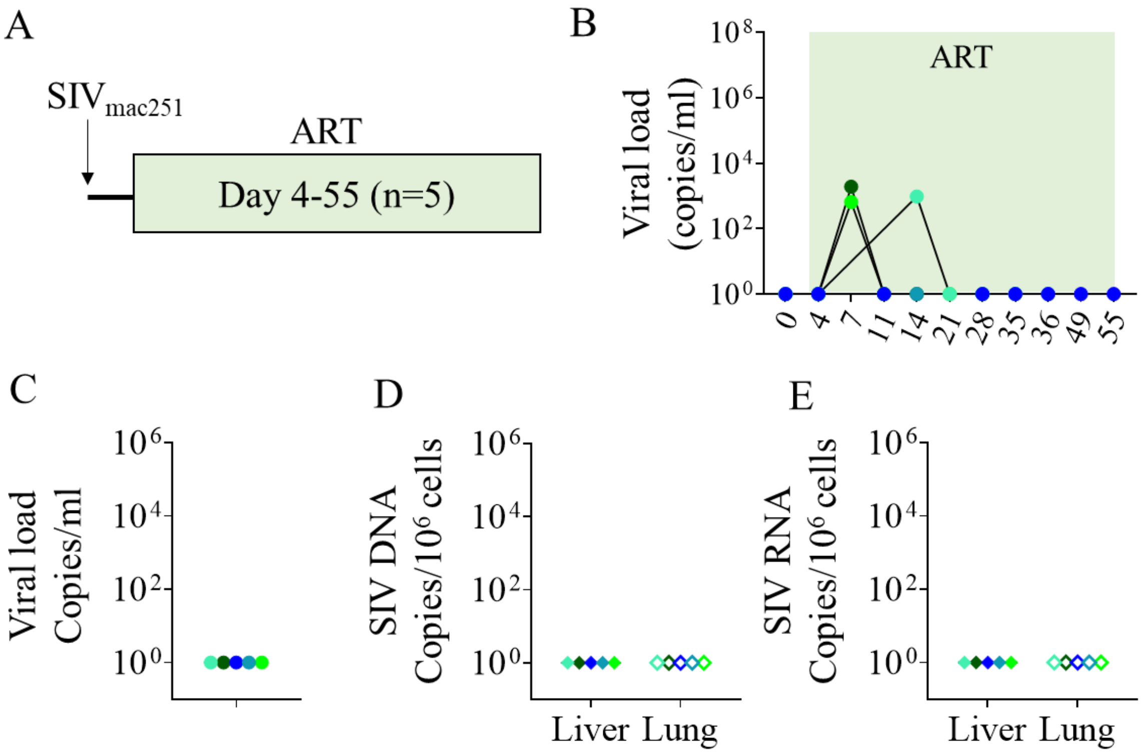

Fig. S1. Frequencies of cell-associated SIV DNA and RNA in the liver and lungs of ART-treated RMs. **A**) Five RMs were infected intravenously with SIVmac251 (20 AID50). At day 4 post-infection, they were treated with Tenofovir (TFV, 20 mg/kg; Gilead) and Emtricitabine (FTC, 40 mg/kg; Gilead) subcutaneously and Raltegravir (RGV, 20 mg/kg; Merck) or Dolutegravir (DTG, 5 mg/kg; ViiV) and Ritonavir (RTV, 20 mg/kg; AbbVie) by oral route (left panel). RMs were sacrificed at day 14, 27, 35, 36 and 55. **B)** The follow-ups of plasma viral loads were performed during the treatment. **C**) Plasma viral loads were quantified for each RM on the day of euthanasia; results are expressed as viral load copies per ml. Frequencies of cell-associated **D**) SIV DNA and **E**) SIV RNA in the liver and lungs were quantified by qRT-PCR in ART-treated RMs. Results are expressed as copies per 10^6 cells. Each color represents one individual. Full circle symbols, blood samples; closed diamond symbols, liver samples; open diamond symbols, lung samples.

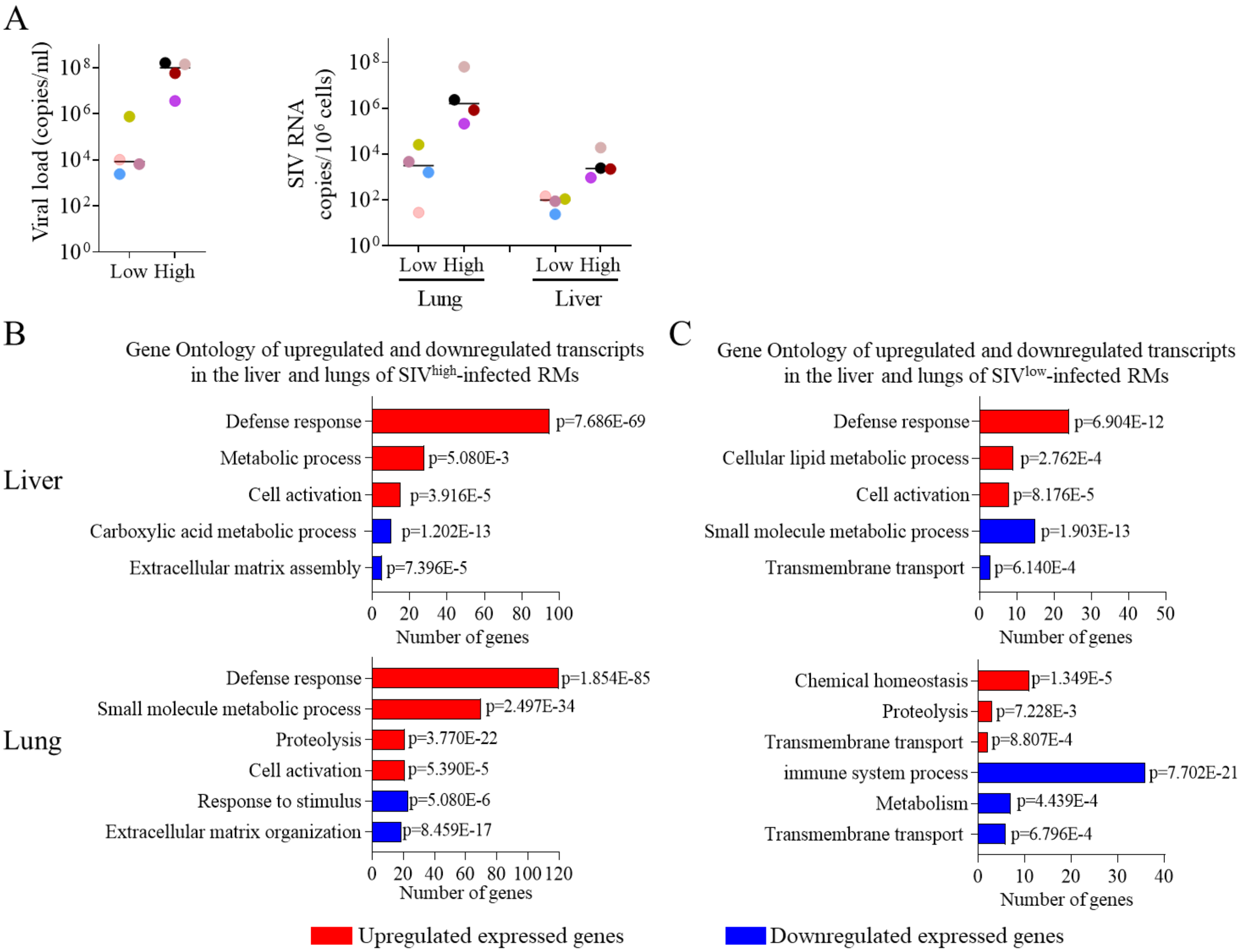

Fig. S2. A) Plasma viral load and cell-associated SIV RNA in the liver and lungs of SIV^{high}-infected RMs and SIV^{low}-infected RMs used for transcriptomic analyses. Functional enrichment analysis of differentially expressed genes in the liver (top panel) and lungs (bottom panel) of **B**) high viremic SIV-infected RMs (SIV^{high}) and **C**) low viremic SIV-infected RMs (SIV^{low}). Functional enrichment analysis were performed using the ToppGene (ToppFun) and g:Profiler webtools. The names of Gene Ontology Biological Process terms (GO_BP) are shown on the y-axes for upregulated (red) and downregulated (blue) genes, and the number of genes involved in each GO_BP are shown on the x-axes.

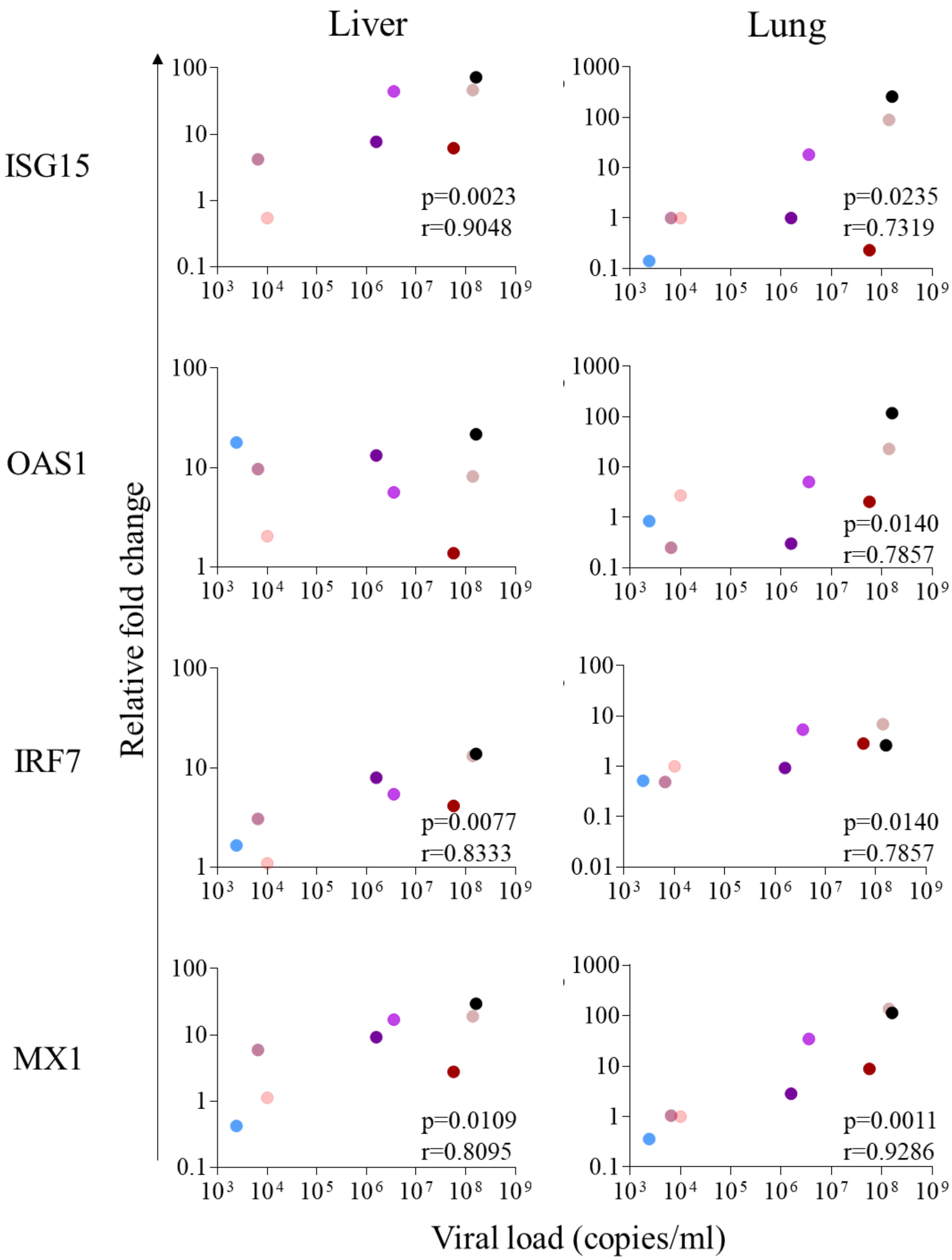

Fig. S3. Positive correlation between mRNA expression of interferon-stimulated genes and viral load. ISG15, OAS1, IRF7 and MX1 mRNA transcripts were quantified by qRT-PCR. Spearman analysis was used for correlations between the levels of viral load and their relative expressions ($2^{-\Delta\Delta Ct}$) in the liver and the lung. The *r* and *p* values are indicated in the figures. Each colored symbol represents one individual (n=8).

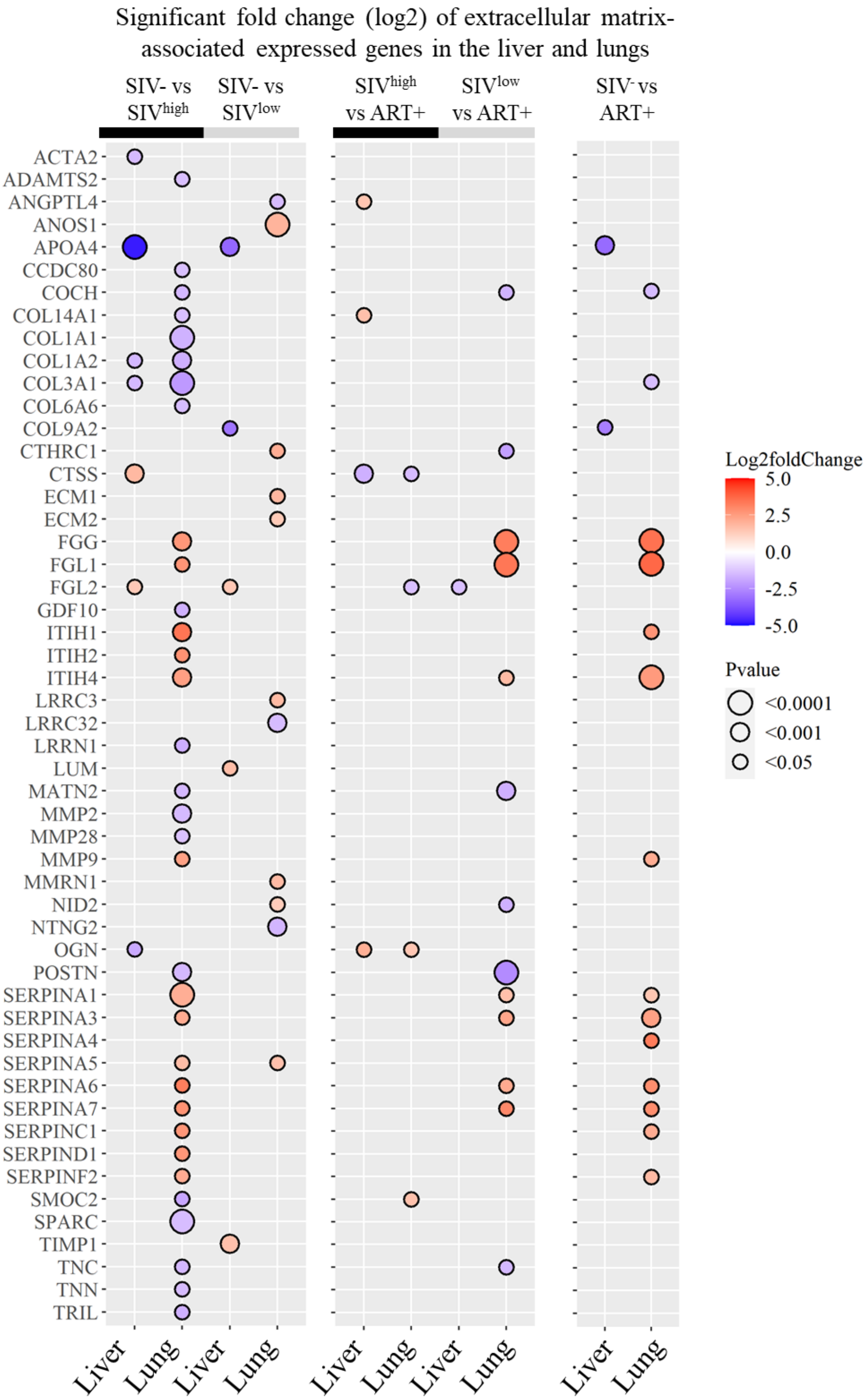

Fig. S4. Significant log2 fold change of transcripts related to extracellular matrix between i) naïve and SIV-infected RMs (left panel), ii) SIV-infected and ART-treated RMs (middle panel), iii) naïve and ART-treated RMs (right panel). The bidirectional color-coded bubbles represent the Log2 fold change z-score, whereas the size of the bubbles indicates the –Log10(P-value) with a threshold of P < 0.0001 for highly significant values. High viremic: SIV^{high}; low viremic: SIV^{low}.

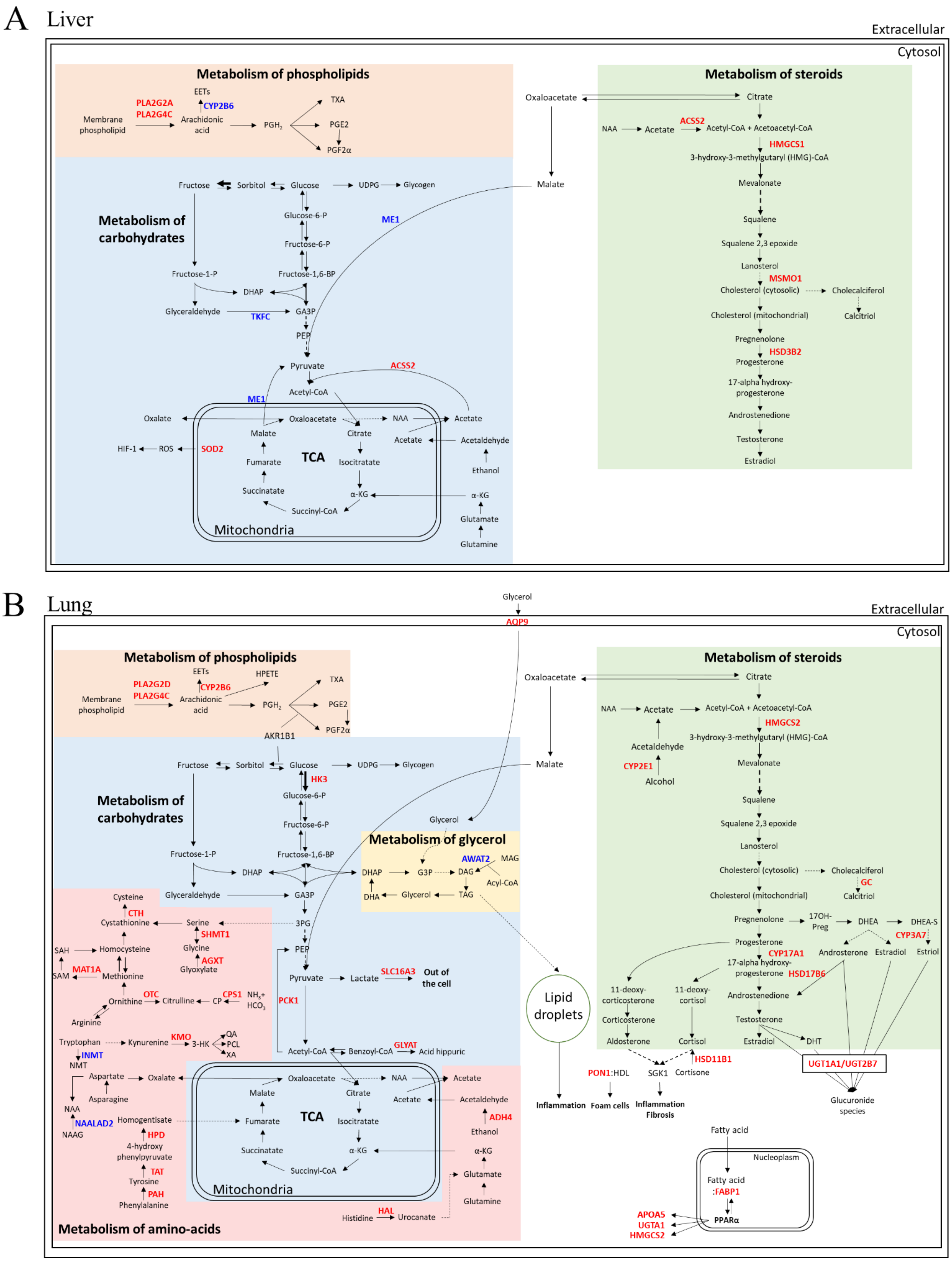

Fig. S5. Schematic metabolic pathways showing upregulated (red) and downregulated (blue) genes **A**) in the liver and **B**) in the lungs of SIV^{high}-infected RMs.

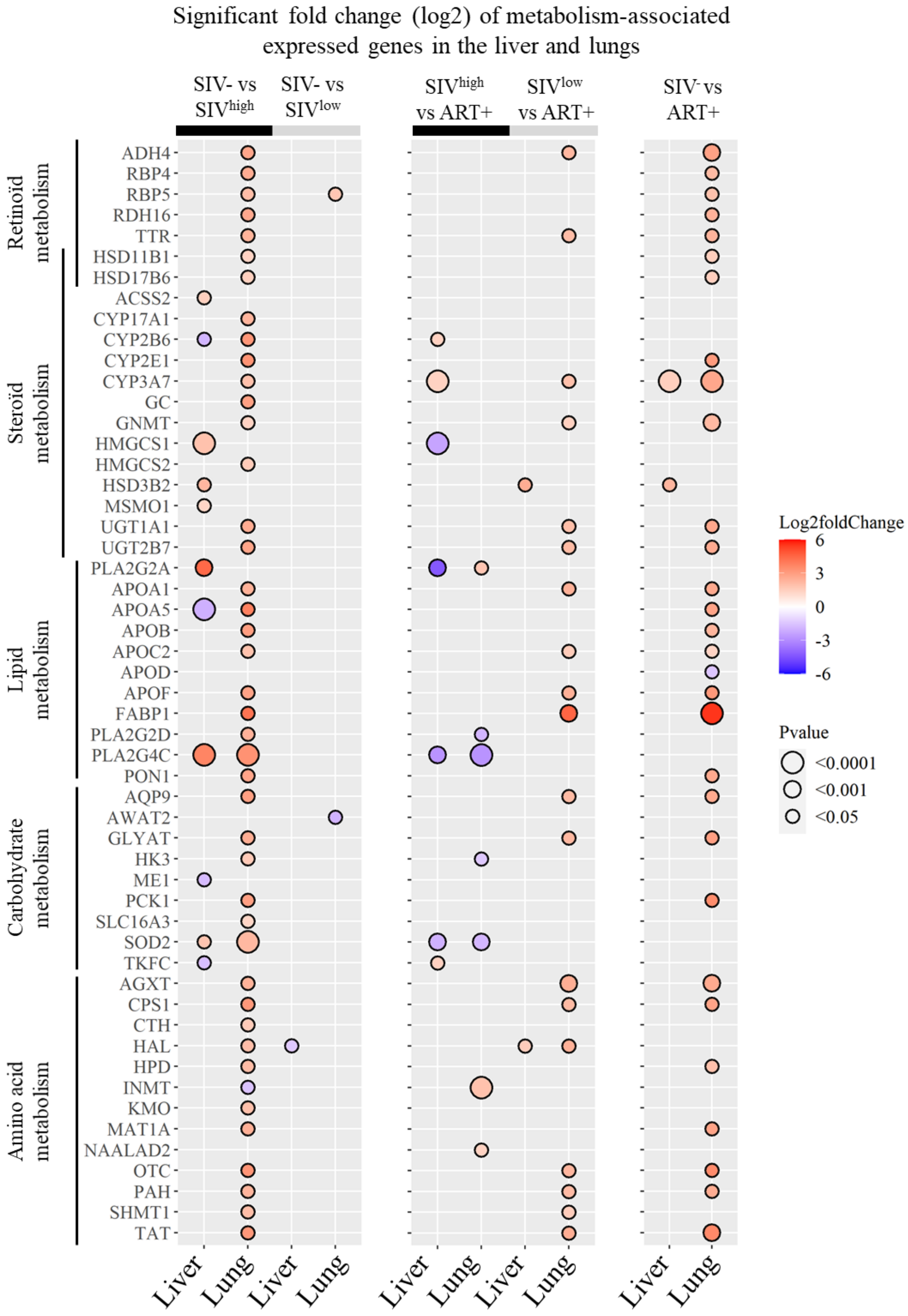

Fig. S6. Significant log2 fold change of transcripts related to metabolism between i) naïve and SIV-infected RMs (left panel), ii) SIV-infected and ART-treated RMs (middle panel), iii) naïve and ART-treated RMs (right panel). The bidirectional color-coded bubbles represent the Log2 fold change z-score, whereas the size of the bubbles indicates the –Log10(P-value) with a threshold of $P < 0.0001$ for highly significant values. High viremic: SIV^{high}; low viremic: SIV^{low}.

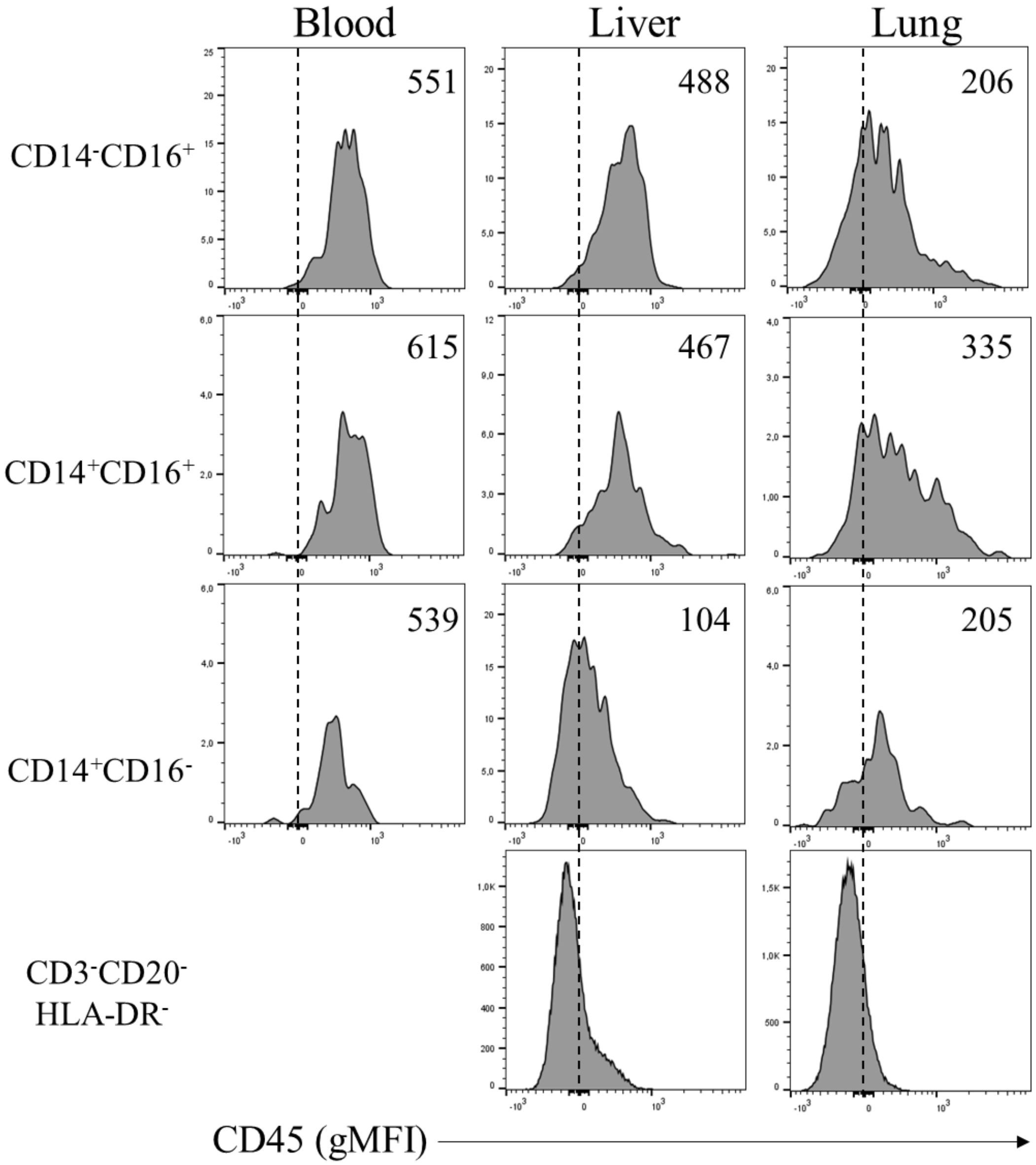

Fig. S7. Geometric mean fluorescence intensity (gMFI) of CD45 marker on CD3$^-$CD20$^-$HLA-DR$^+$CD11b$^+$ expressing CD14$^-$CD16$^+$, CD14$^+$CD16$^+$, CD14$^+$CD16$^-$ cells and CD3$^-$CD20$^-$HLA-DR$^-$ cells from peripheral blood, liver, and lungs.

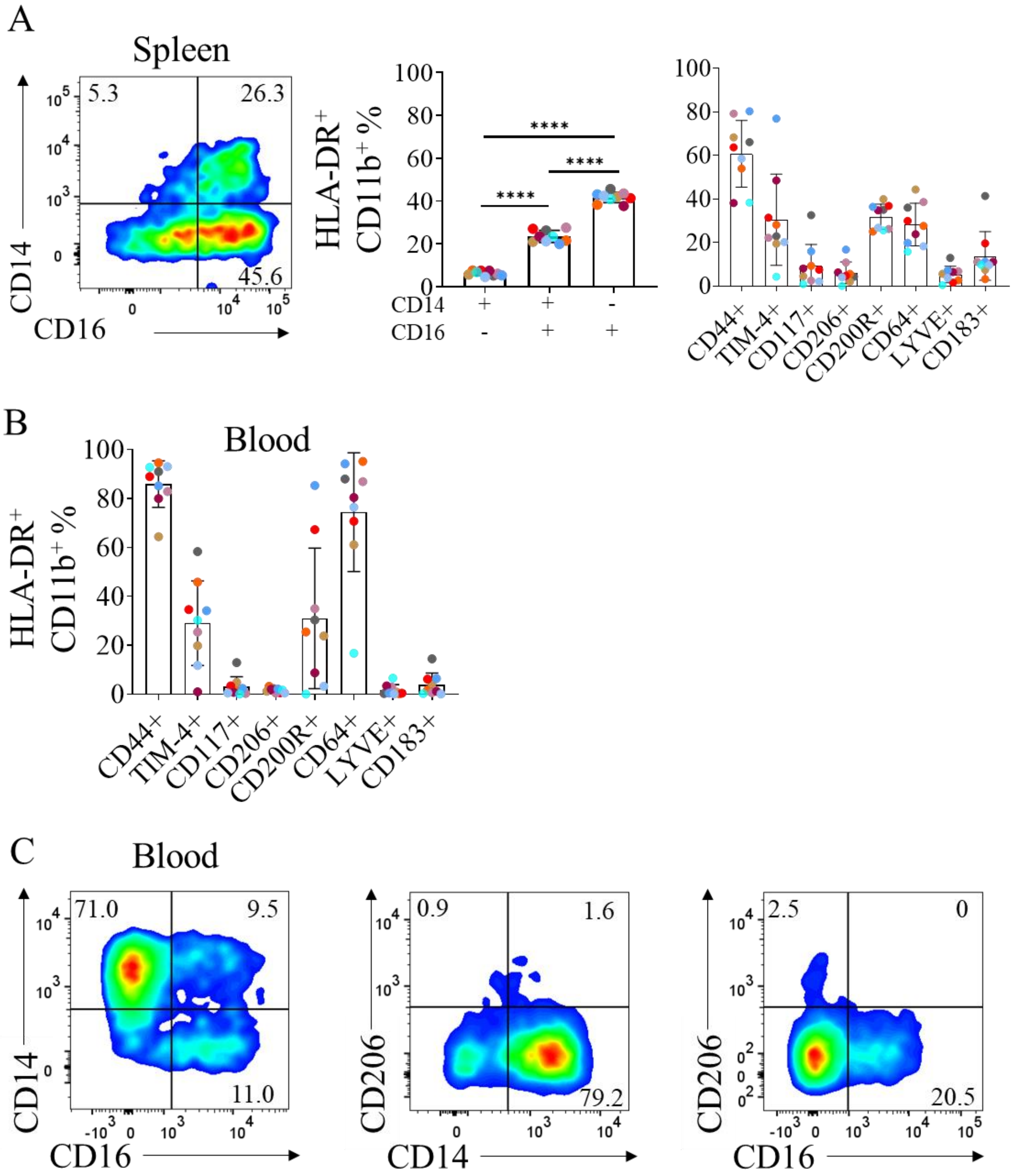

Fig. S8. Expression of TRM markers in the spleen and blood. **A**) Left, representative dot plots depicting the expression of CD14 and CD16 among CD3$^-$CD20$^-$HLA-DR$^+$CD11b$^+$ splenic cells are shown. Histograms show the percentages of classical (CD14$^+$CD16$^-$), intermediate (CD14$^+$CD16$^+$), non-classical (CD14$^-$CD16$^+$) cells and TRM markers. **B**) Histogram shows the percentages of CD3$^-$CD20$^-$HLA-DR$^+$CD11b$^+$ cells expressing TRM markers in the blood. Each colored symbol represents one individual. **C**) Representative dot plots depicting the expression of CD14 versus CD16, CD206 versus CD14 and CD206 versus CD16 in CD3$^-$CD20$^-$HLA-DR$^+$CD11b$^+$ blood cells.

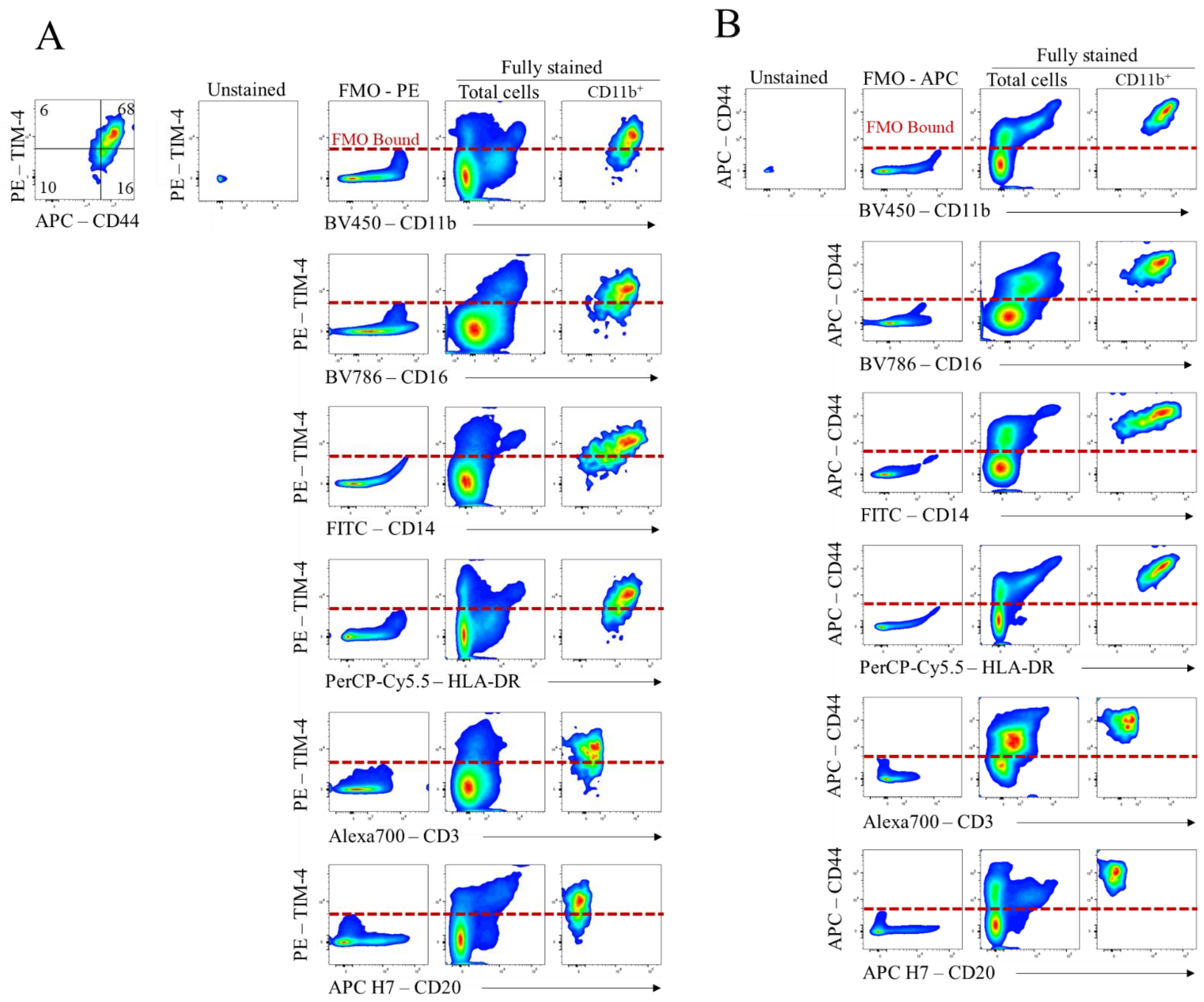

Fig. S9. Fluorescence minus one (FMO) control in pulmonary cells for determining **A**) PE and **B**) APC positive signals in the lung sample. **A**) Left, representative dot plot depicting the expression of TIM-4 (PE) and CD44 (APC) in $CD3^{-}CD20^{-}HLA\text{-}DR^{+}CD11b^{+}$ cells. Unstained cells, PE-FMO control and fully stained samples are shown after compensation in total cells and in $CD3^{-}CD20^{-}HLA\text{-}DR^{+}CD11b^{+}$ cells. **B**) Unstained cells, APC-FMO control and fully stained samples are shown after compensation in total cells and in $CD3^{-}CD20^{-}HLA\text{-}DR^{+}CD11b^{+}$ cells. FMO against each staining is shown (anti-CD11b-BV450, anti-CD16-BV786, anti-CD14-FITC, anti-HLA-DR-PerCP-Cy5.5, anti-CD3-Alexa700, anti-CD20-APC.H7).

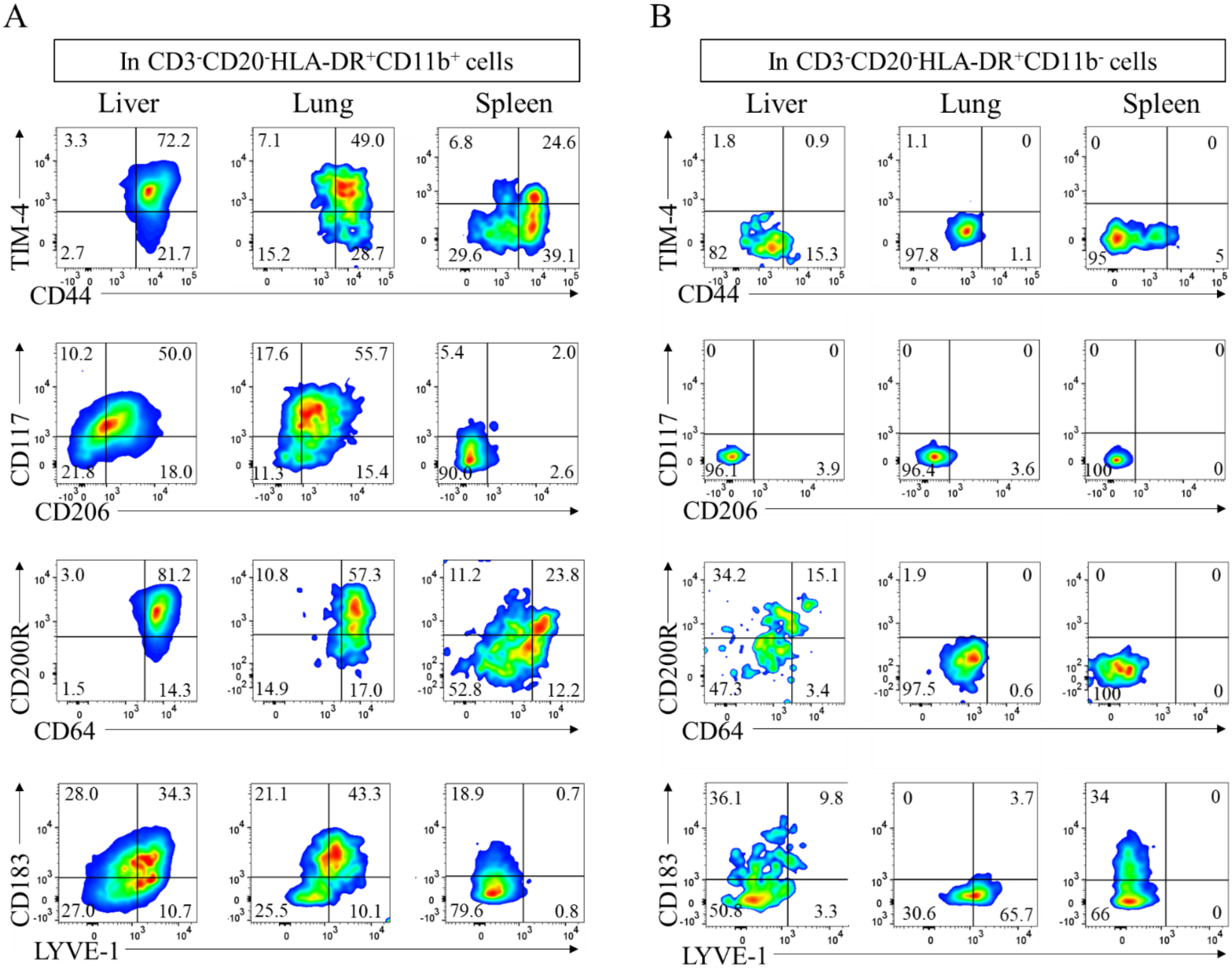

Fig. S10. Phenotypes of HLA-DR$^+$CD11b$^+$ myeloid cells. Representative dot plots depicting the expression of TIM-4 versus CD44, CD117 versus CD206, CD200R versus CD64, CD183 versus LYVE-1 in **A**) CD3$^-$CD20$^-$HLA-DR$^+$CD11b$^+$ cells and **B**) CD3$^-$CD20$^-$HLA-DR$^+$CD11b$^-$ cells from the liver, lung, and spleen of an SIV-infected RM.

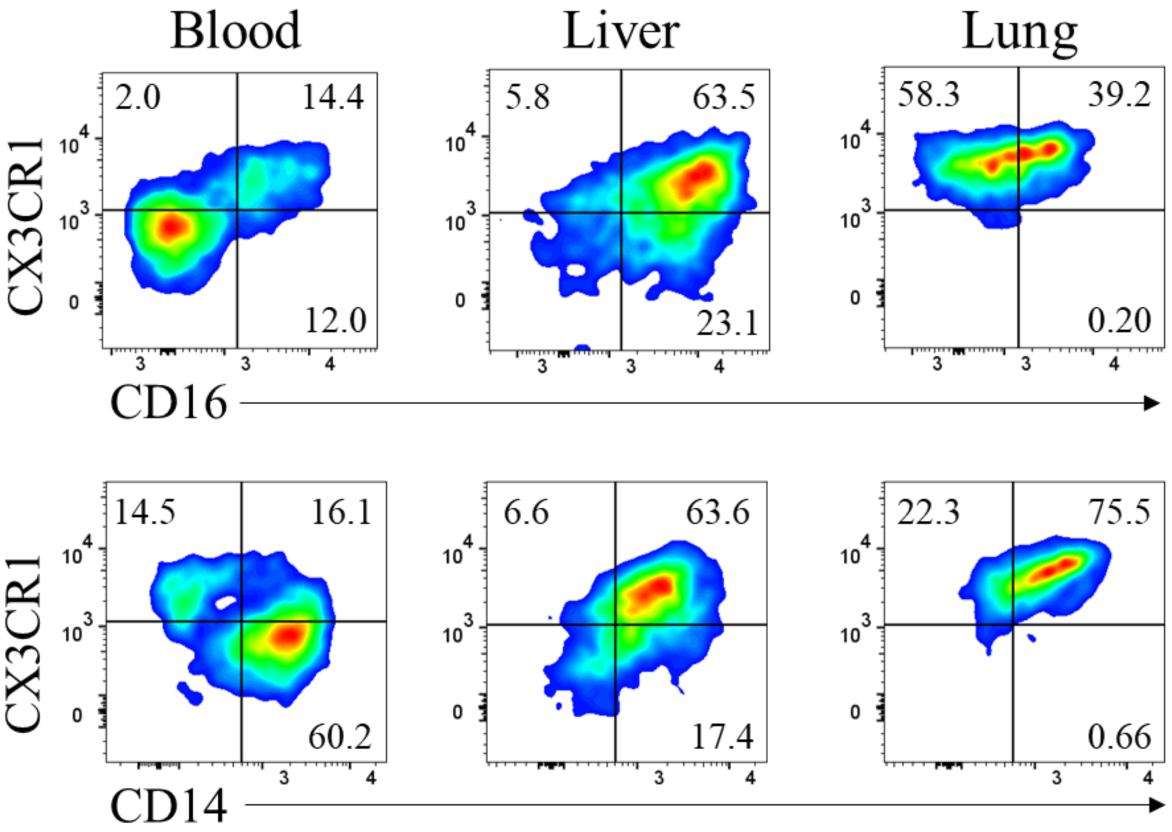

Fig. S11. Dot plots show the expression of CX3CR1 against either CD16 (top panels) or CD14 (bottom panels) in CD3$^-$CD20$^-$HLA-DR$^+$CD11b$^+$ cells from the blood, liver, and lung of an SIV-infected RM.

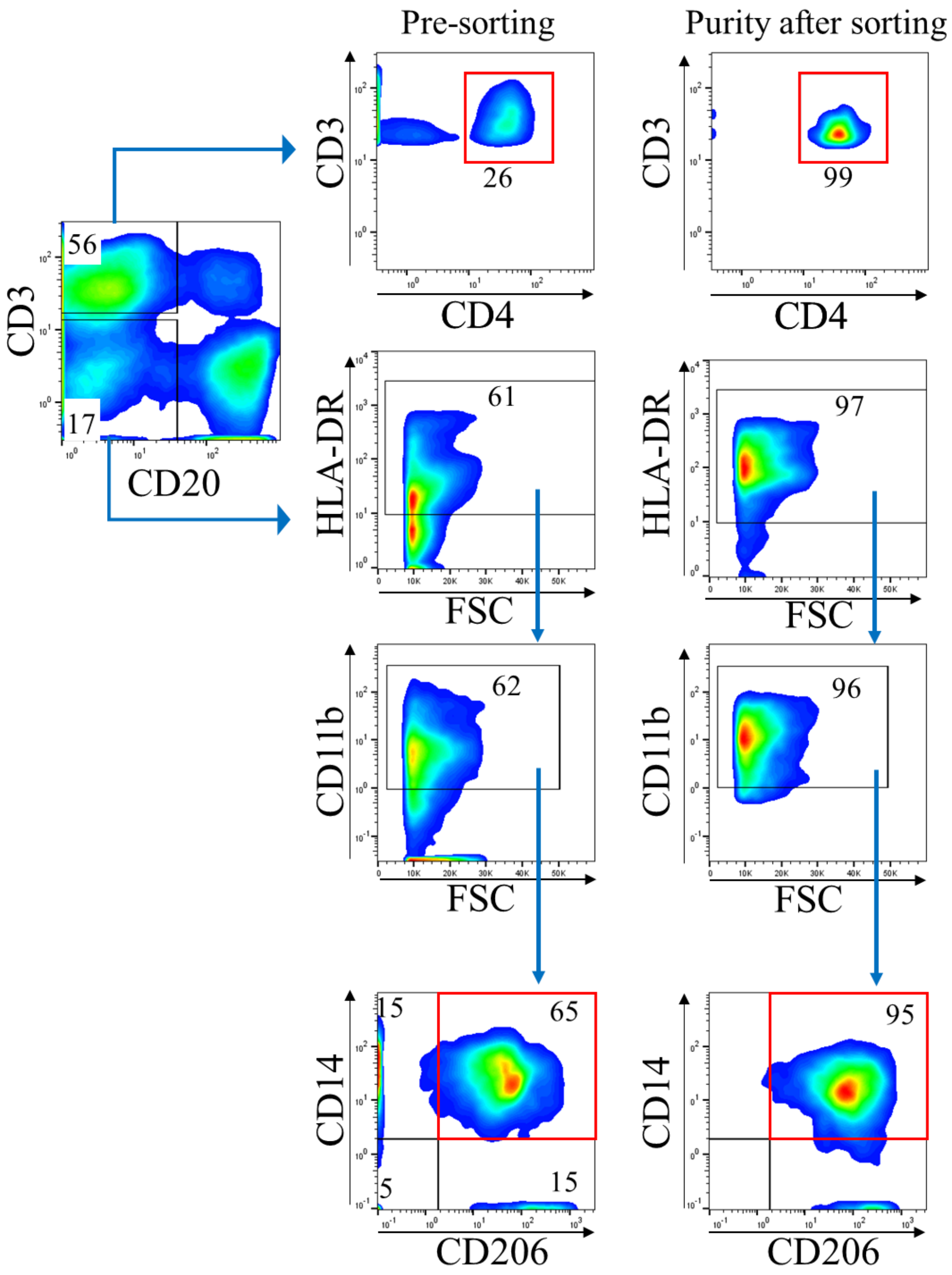

Fig. S12. Cell purity after cell sorting of hepatic CD4$^+$ T and myeloid cells. Representative dot plots depicting cells before and after cell sorting. Cell purities are indicated and higher than 95% for all sorted subsets.

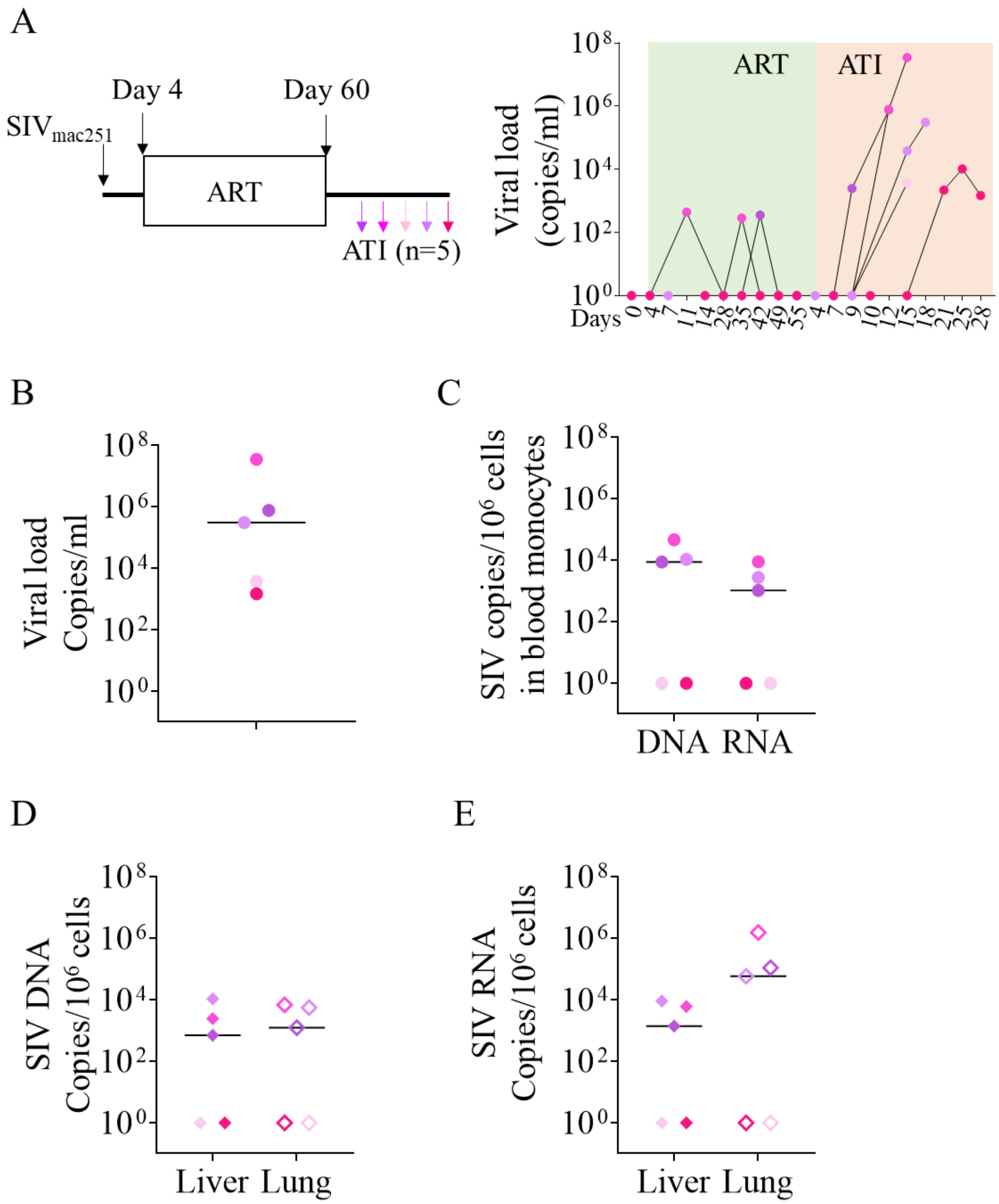

Fig. S13. Viral rebound after ART interruption. **A**) After 60 days of treatment, five RMs were sacrificed at days 12, 15, 18 and 28 after ART interruption (ATI) (left panel). The follow up of plasma viral loads were performed during the treatment and after ATI (right panel). **B**) Viral loads quantified on the day of euthanasia; results are expressed as viral load copies per ml. **C**) Frequencies of SIV DNA and RNA were quantified by qRT-PCR in sorted $CD14^+$ blood monocytes of RMs after ATI. Frequencies of cell-associated **D**) SIV DNA and **E**) SIV RNA were quantified by qRT-PCR in the liver and lung of RMs after ATI. Results are expressed as copies per 10^6 cells. Each color represents one individual. Full circle symbols, blood samples; closed diamond symbols, liver samples; open diamond symbols, lung samples.
